# Hyperadhesive von Willebrand Factor Promotes Extracellular Vesicle-Induced Angiogenesis: Implication for LVAD-Induced Bleeding

**DOI:** 10.1016/j.jacbts.2021.12.005

**Published:** 2022-03-28

**Authors:** Mengchen Yang, Katie L. Houck, Xinlong Dong, Maria Hernandez, Yi Wang, Sriram S. Nathan, Xiaoping Wu, Vahid Afshar-Kharghan, Xiaoyun Fu, Miguel A. Cruz, Jianning Zhang, Angelo Nascimbene, Jing-fei Dong

**Affiliations:** a Bloodworks Research Institute, Seattle, Washington, USA; b Department of Urology, Tianjin Medical University General Hospital, Tianjin, China; c Center for Advanced Heart Failure, University of Texas at Houston, Houston, Texas, USA; d Department of Pathology, University of Washington School of Medicine, Seattle, Washington, USA; e Division of Internal Medicine, Department of Pulmonary Medicine, MD Anderson Cancer Center, University of Texas, Houston, Texas, USA; f Cardiovascular Research Section, Department of Medicine, Baylor College of Medicine; g Center for Translational Research on Inflammatory Diseases, Michael E. DeBakey VA Medical Center, Houston, Texas, USA; h Department of Neurosurgery, Tianjin Medical University General Hospital, Tianjin, China; i Division of Hematology, Department of Medicine, University of Washington School of Medicine, Seattle, Washington, USA

**Keywords:** angiogenesis, extracellular vesicles, left ventricular assist devices, platelets, shear stress, von Willebrand factor, AVS, aortic vascular segment, ADAMTS-13:Ag, ADAMTS-13 antigen, aVWS, acquired von Willebrand syndrome, EC, endothelial cell, EV, extracellular vesicle, EVFP, extracellular vesicle–free plasma, GI, gastrointestinal, GOF, gain of function, GP, glycoprotein, GPM, growth factor-poor medium, GRM, growth factor-rich medium, HSS, high shear stress, LVAD, left ventricular assist device, pEV, extracellular vesicle from von Willebrand factor-activated platelets, PS, phosphatidylserine, SIPA, shear-induced platelet aggregation, ULVWF, ultra-large von Willebrand factor, VEGF, vascular endothelial growth factor, VWF, von Willebrand factor, VWF:Ag, von Willebrand factor antigen, VWF:CB, von Willebrand factor binding to collagen, VWF:pp, von Willebrand factor propeptide

## Abstract

Bleeding associated with left ventricular assist device (LVAD) implantation has been attributed to the loss of large von Willebrand factor (VWF) multimers to excessive cleavage by ADAMTS-13, but this mechanism is not fully supported by the current evidence. We analyzed VWF reactivity in longitudinal samples from LVAD patients and studied normal VWF and platelets exposed to high shear stress to show that VWF became hyperadhesive in LVAD patients to induce platelet microvesiculation. Platelet microvesicles activated endothelial cells, induced vascular permeability, and promoted angiogenesis in a VWF-dependent manner. Our findings suggest that LVAD-driven high shear stress primarily activates VWF, rather than inducing cleavage in the majority of patients.

## Highlights

• VWF in patients on LVAD supports was hyperadhesive, activated platelets, and generated platelet-derived extracellular vesicles.
• Extracellular vesicles from LVAD patients and those from shear-activated platelets promoted aberrant angiogenesis in a VWF-dependent manner.
• The activated VWF exposed the A1 domain through the synergistic actions of oxidative stress and HSS generated in LVAD-driven circulation.

## Visual Abstract

**Figure:**
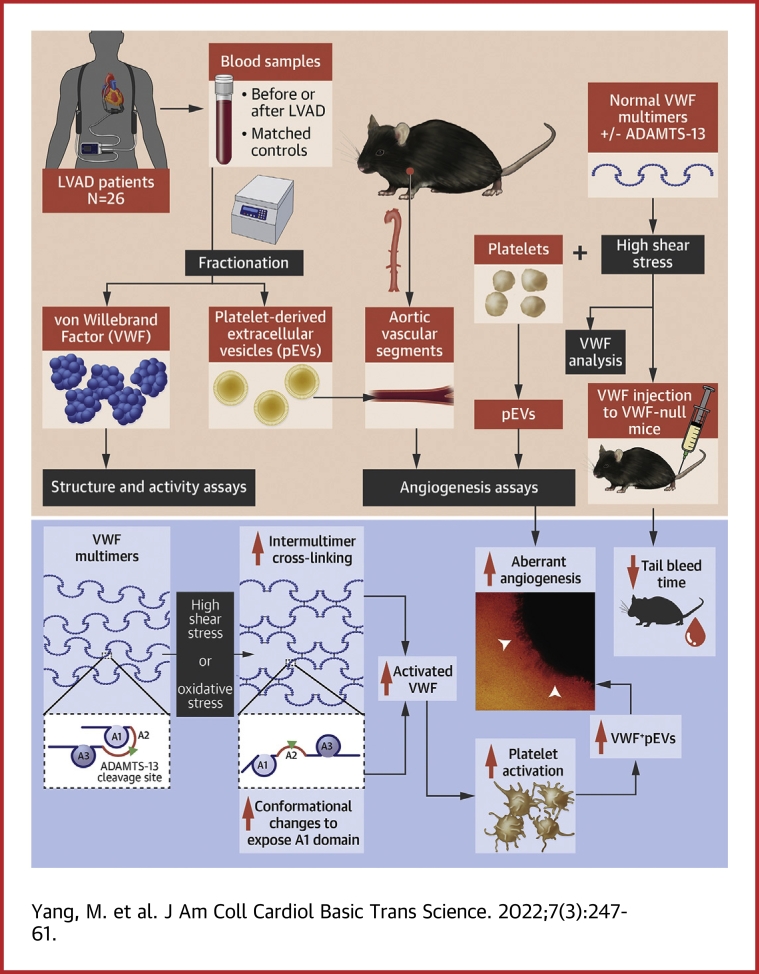

Left ventricular assist devices (LVADs) support cardiac output for patients with end-stage heart failure and have significantly improved these patients’ survival. However, device-related complications remain common and often result in poor outcomes for patients.^1^, ^2^, ^3^ Severe bleeding is found in 11% to 30% of patients on axial-flow LVADs^4^, ^5^, ^6^, ^7^, ^8^, ^9^, ^10^ and remains common in patients on centrifugal-flow LVADs, which drastically reduce the thrombotic complications associated with axial-flow LVADs.^11^ Gastrointestinal (GI) bleeding is most common and is found at the site of angiodysplasia in more than 50% of cases.^12^^,^^13^ Post-LVAD GI bleeding has been shown to be reduced in patients receiving angiotensin-converting enzyme inhibitors or angiotensin receptor blockage after LVAD implantation,^14^^,^^15^ suggesting that angiogenesis is a causal factor for LVAD-induced bleeding.

Nearly all LVAD patients lose large VWF multimers in a condition called acquired von Willebrand syndrome (aVWS). aVWS develops soon after LVAD implantation, resolves rapidly after LVAD explantation, and is not observed in heart transplant recipients,^16^^,^^17^ suggesting that it is caused by hydrodynamic changes in the LVAD-driven blood flow.

VWF is synthesized in megakaryocytes and endothelial cells (ECs) as a single-chain propolypeptide that dimerizes through C-terminal disulfide bonds and then multimerizes through N-terminal disulfide bonds. Each VWF monomer contains the binding sites for glycoprotein (GP) Ibα and collagen in the A1 and A3 domains, respectively; the Y^1605^-M^1606^ peptide bond cleaved by ADAMTS-13 in the A2 domain; and an integrin-binding RGD in the C-domain. Newly synthesized VWF multimers are either constitutively released (smaller multimers) or stored in the Weibel-Palade bodies of ECs and the α-granules of platelets, where VWF is enriched in ultra-large multimers (ULVWF) that are intrinsically hyperactive in binding platelets and endothelial cells.^18^^,^^19^ Upon release, these ULVWF multimers are anchored to the endothelium and rapidly but partially cleaved by ADAMTS-13.^19^, ^20^, ^21^, ^22^ The cleavage converts constitutively hyperadhesive ULVWF multimers^23^^,^^24^ into plasma VWF multimers that bind circulating platelets poorly but can be activated to increase their adhesive activity by high shear stress (HSS).^23^^,^^24^

HSS at similar levels has been shown to reduce VWF adhesive activity by exposing the cryptic Tyr^1605^-Met^1606^ scissile bond in the A2 domain to facilitate VWF cleavage and also to activate plasma VWF by exposing the GP Ibα-binding site in the A1 domain.^25^ These 2 opposing processes have almost exclusively been studied individually using in vitro techniques, raising the critical question of how the 2 processes reach equilibrium in vivo to maintain adequate hemostasis without causing thrombosis or bleeding. LVAD-induced aVWS has been widely attributed to the excessive cleavage of VWF by ADAMTS-13,^26^ but the role of shear-induced VWF activation is not known.

We investigated VWF cleavage and activation under LVAD-driven circulation by: 1) studying longitudinal samples from LVAD patients; 2) identifying shear-induced structural changes of VWF in vivo and in vitro; and 3) dissecting the interplay between extracellular vesicles (EVs) and hyperadhesive VWF in promoting the aberrant angiogenesis that could be the cause of the angiodysplasia found in the mucosal tissue of LVAD patients.

## Methods

### Patients

Blood samples were collected from 26 patients admitted to the Center for Advanced Heart Failure, the University of Texas Houston Health Science Center. All patients had New York Heart Association functional class IV symptoms and received HeartMate II LVADs (Abbott Laboratories). Patients with malignancies and autoimmune disease were excluded. All patients received standard post-implantation antithrombotic therapy: aspirin (81 mg/d), warfarin with a targeted international normalized ratio of 2 to 3, and dipyridamole (75 mg, 3 times daily). While some patients reported as regular smokers at the baseline, none reported smoking post-implantation because all patients were instructed to stop smoking as part of their post-LVAD care.

Blood samples (20-30 mL) were collected at the time of admission (baseline), at discharge after LVAD implantation (mean 28 days), and at the time when a patient was readmitted for an adverse clinical event (bleeding or thrombosis) between discharge and the 3-month follow-up visit. The blood samples at clinical events were collected at presentation before any treatment was administered. Patient samples were coded before analyses to minimize experimental bias. Age- and gender-matched healthy volunteers were studied as control subjects. This study was approved by the review boards on human subject research of the University of Texas and the Bloodworks Research Institute.

The data from patients with GI bleeding and thrombosis are presented separately in the results from directly analyzing the plasma samples of patients. For experiments in which patient EVs were tested for endothelial permeability, angiogenesis, and thrombus formation, the data from patients with thrombosis and those with bleeding were combined.

### 3-dimensional Matrigel angiogenesis assay

The aortas from C57BL/6J mice (23-25 weeks old, 50% male; The Jackson Laboratory) euthanized under anesthesia were collected and dissected into 2-mm aortic vascular segments (AVSs). The AVSs were incubated in serum-free Dulbecco's Modified Eagle Medium (Thermo Fisher Scientific) overnight at 37°C and then placed in microwells coated with Matrigels (Corning Life Sciences) containing growth factor–poor medium (GPM) (Supplemental Figure 1). They were then incubated at 37 °C for 30 minutes before being covered with Matrigel and cultured in Dulbecco's Modified Eagle Medium containing 5% of fetal bovine serum for 14 days at 37 °C with daily additions of EVs (1 × 10^6^/mL) (Supplemental Methods) or EV-free plasma (EVFP). In a subset of experiments, the AVSs were treated with EVs in the presence of a polyclonal VWF antibody (Cat #: ab6994; Abcam). AVSs cultured in GPM Matrigels alone and those in growth factor–rich medium (GRM) Matrigels without EVs were tested as control samples. AVSs were also cultured with EVs from control platelets exposed to 110 dynes/cm^2^ of HSS (Supplemental Methods). The AVSs were observed daily under an inverted-stage microscope (Olympus IX81) to quantify the number and length of vascular sprouts. After 14 days, the AVSs were collected, fixed in 5% of formalin, and sectioned for CD31 immunocytochemistry using a monoclonal antibody (Cat #: ab28364; Abcam).

### VWF assays

We used multiple assays to comprehensively evaluate VWF, including: 1) VWF antigen (VWF:Ag) and VWF propeptide (VWF:pp) measured by enzyme-linked immunosorbent assay (Cat #: ab223864 [Abcam] and CS-MW1939 [Cell Sciences]); 2) thiols exposed on the surface of VWF multimers (Supplemental Methods); 3) ristocetin-induced platelet agglutination (RIPA) (Helena Laboratories); 4) VWF binding to collagen (VWF:CB) (Supplemental Methods); 5) platelet adhesion under flow conditions (Supplemental Methods); 6) VWF multimers using nonreducing sodium dodecyl sulfate–agarose gel electrophoresis (Supplemental Methods);^27^ 7) shear-induced platelet aggregation (SIPA) (Supplemental Methods); 8) the exposure of the A1 domain on VWF using immunoprecipitation (Supplemental Methods); 9) endothelial permeability (Supplemental Methods); and 10) ADAMTS-13 antigen (ADAMTS-13:Ag) using enzyme-linked immunosorbent assay (Cat #: ab234559; Abcam) and cleaving an exogenous VWF peptide measured using a FRET (fluorescence resonance energy transfer) assay (Diapharma).

### Mouse experiments

Wild-type C57BL/6J mice and VWF-deficient mice on C57BL/6J background (The Jackson Laboratory) were infused with VWF multimers purified from human plasma cryoprecipitate or an equal volume of saline through the tail vein. VWF was tested at 30 μg/mL to be consistent with the amount found in LVAD patients and was infused before and immediately after exposure to 110 dynes/cm^2^ of HSS for 5 minutes at 37 °C. The hemostasis of mice was evaluated using tail vein bleeding (Supplemental Methods).

### Statistical analysis

All data are presented as mean ± SEM for continuous variables or as percentages for categorical variables using SigmaPlot V11.2 (Systat). The Shapiro-Wilk normality test was performed to determine if the data followed a normal distribution. Student’s *t* test was used to compare quantitative variables between 2 groups. Both paired and unpaired methods were used in the analyses, depending on the nature of the data. One-way analysis of variance or 1-way analysis of variance on ranks was used to compare more than 2 groups with Bonferroni post hoc test for multiple pairwise comparisons. A *P* value <0.05 was considered statistically significant.

## Results

Among the 26 patients (Table 1), 4 developed GI bleeding, 1 developed pump thrombosis, and 2 developed stroke in the first 3 months post-LVAD (pump thrombosis and stroke were grouped together). This study was designed to mechanistically define LVAD-induced changes of VWF reactivity and was not powered to associate VWF changes to clinical events because of its small sample size.

**Table 1:** Information on Patients Included in the Study

Age, y	64.3 ± 8.6
Female	12 (46.0)
Destination therapy	21 (80.0)
Patients with adverse events	7 (26.9)
Thrombosis	3 (42.9)
GI bleeding	4 (57.1)
MAP, mm Hg	85.3 ± 9.6
Laboratory findings	
Hemoglobin, g/dL	11.6 ± 3.5
Platelet counts (×10^3^/μL)	260.2 ± 115.8
INR	2.1 ± 1.0
Albumin, g/dL	3.50 ± 0.28
Creatinine, mg/dL	1.1 ± 0.3
WBC (×10^3^/μL)	7.6 ± 3.1
Bilirubin total, mg/dL	1.0 ± 0.3
Bilirubin indirect, mg/dL	0.4 ± 0.3
LVAD setting	
RPM	8,869 ± 682
PI	5.23 ± 2.00
PW	5.90 ± 1.51

Values are mean ± SD or n (%).

GI = gastrointestinal; INR = international normalized ratio; LVAD = left ventricular assist device; MAP = mean arterial pressure; PI = pulsatility index; PW = power; RPM = revolution per minute; WBC = white blood cell count.

### Patient plasma increased vascular permeability

**Figure 1:**
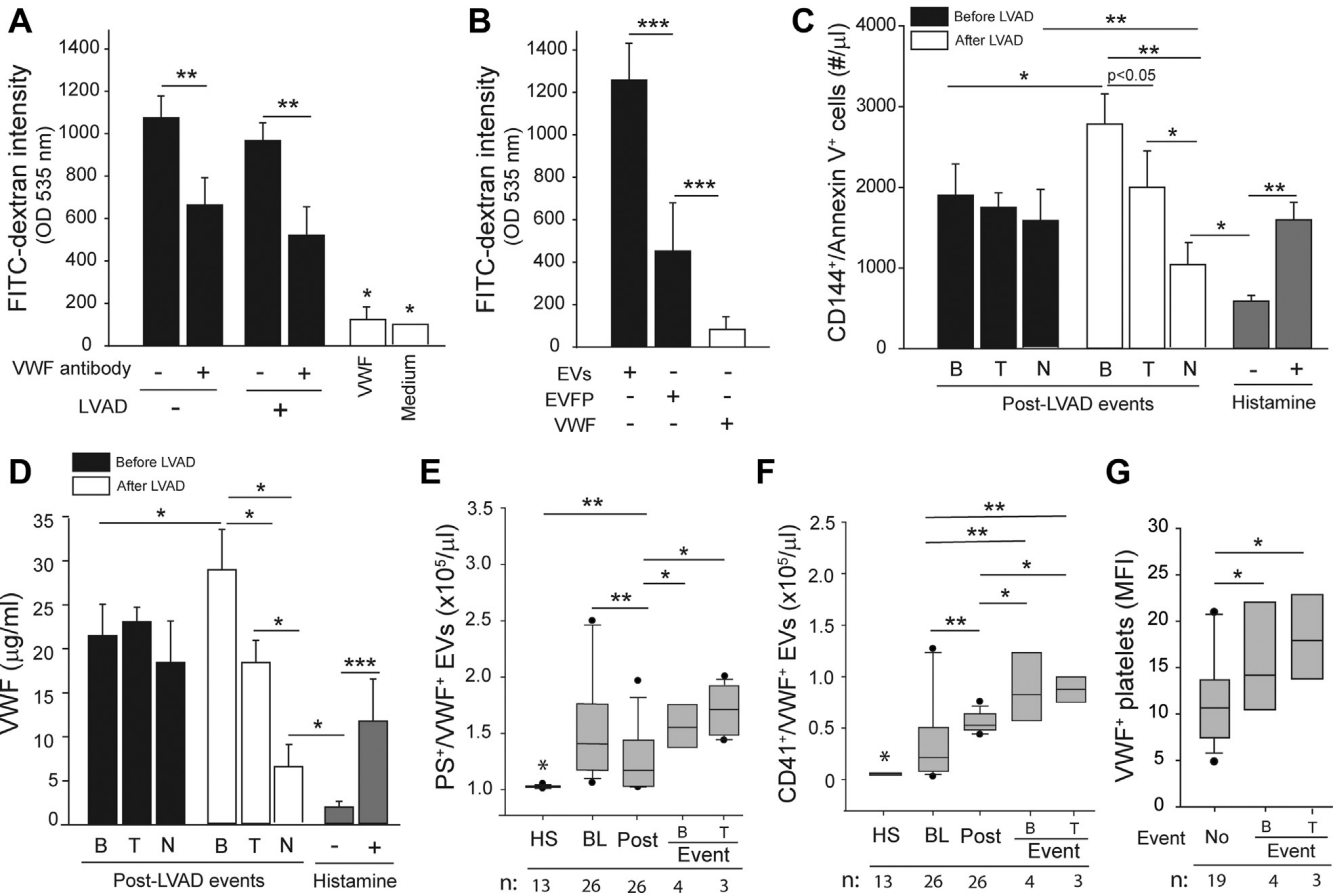
VWF Mediated EV-Induced Vascular Permeability **(A)** Endothelial permeability was induced by patient plasma collected at discharge but not by purified von Willebrand factor (VWF) at 30 μg/mL (n = 26 for patient plasma and n = 6 for VWF). The permeability was blocked by the VWF-blocking antibody. **(B)** Extracellular vesicles (EVs) but not extracellular vesicle–free plasma (EVFP) induced endothelial permeability (n = 26). **(C)** Phosphatidylserine (PS) expression and **(D)** VWF released from cultured endothelial cells stimulated with plasma collected before and after left ventricular assist device (LVAD) implants. The post-LVAD data were stratified into bleeding (B) (n = 4), thrombosis (T) (n = 3), and no complication (N) (n = 19) and with baseline values subtracted. Histamine-treated endothelial cells (25 μM, 20 minutes at 37 °C) served as the control samples. EVs stained with anti-VWF antibody together with either **(E)** annexin V or **(F)** anti-CD41a antibody in plasma collected from healthy subjects (HS) and patients at baseline (BL), at discharge (Post), and at clinical events of bleeding (B) or thrombosis (T). **(G)** VWF on platelets from LVAD patients. For **E to G**, the sample sizes are provided at the bottom of the figures. All data were analyzed using 1-way analysis of variance (∗*P <* 0.05, ∗∗*P <* 0.01, ∗∗*P <* 0.001). MFI = mean fluorescence intensity; OD = optical density.

Plasma from patients before and after LVAD implantation induced significant endothelial permeability (Figure 1A). The permeability was blocked by a VWF-blocking antibody (5 μg/mL) that we have recently shown to block EV-induced endothelial permeability in a trauma setting,^28^ but it was not induced by purified VWF at 30 μg/mL. When patient plasma was fractionated and tested separately, EVs were significantly more active than the EVFP in inducing permeability (Figure 1B). Plasma samples collected from patients at baseline activated cultured ECs to express phosphatidylserine (PS) (CD144^+^/Annexin V^+^) (Figure 1C) and to release VWF (Figure 1D). Furthermore, the plasma samples collected from patients at the post-LVAD bleeding events activated more endothelial cells to express PS and to release VWF (Figures 1C and 1D), whereas those from patients with thrombosis induced permeability at similar levels to pre-LVAD samples. In contrast, plasma samples from patients without clinical events had less EC-activating activity compared with their pre-LVAD samples.

The levels of total VWF-bound EVs (VWF^+^/PS^+^) were significantly higher in patients at baseline than in the control subjects (Figure 1E). They were reduced moderately post-LVAD but increased again at comparable levels between patients with bleeding and those with thrombosis. In contrast, CD41^+^/VWF^+^ EVs from VWF-activated platelets (pEVs) were significantly increased after LVAD implants and increased further at clinical events (Figure 1F), accounting for 17.9% ± 8.2% of VWF^+^ EVs in pre-LVAD samples and 51.3% ± 16.1% in post-LVAD samples (paired *t* test, *P <* 0.001), suggesting a significant increase in platelet microvesiculation in LVAD-driven blood flow. No significant difference was detected in the levels of pEVs between patients with bleeding and those with thrombosis. Consistent with these observations, more VWF was detected on platelets collected at bleeding or thrombotic events than on those collected outside of these events (Figure 1G). Together, these data suggest that EVs in plasma from patients at the baseline activated ECs to increase permeability and that this activity increased in patients with bleeding and was associated with pEVs, which were significantly increased in post-LVAD samples.

### EVs promoted VWF-dependent angiogenesis

**Figure 2:**
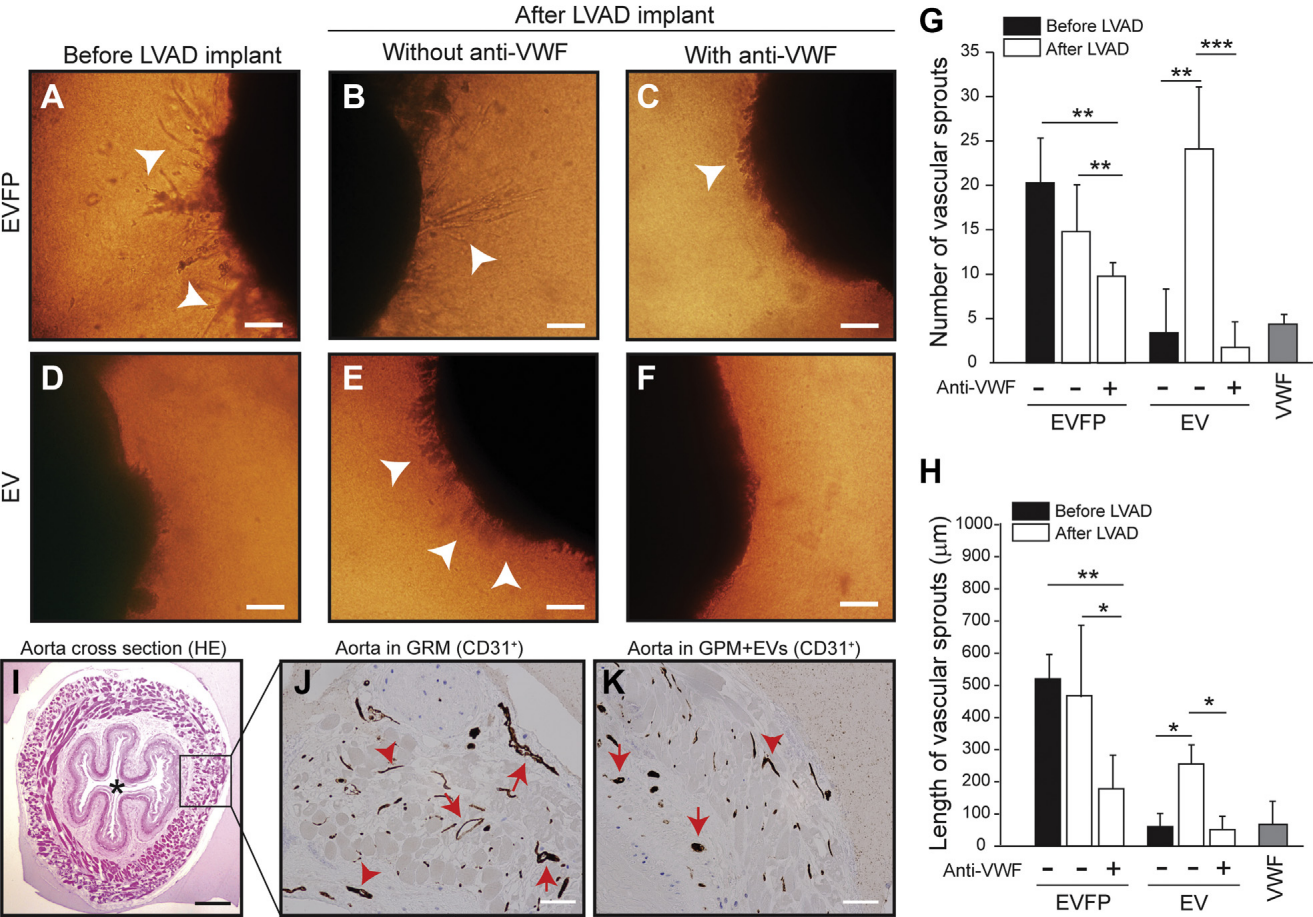
EVs Promoted VWF-Dependent Angiogenesis **(A to F)** Representative images of angiogenesis from aortic vascular segment (AVSs) cultured for 14 days in growth factor–poor medium (GPM) supplemented with EVFP or EVs from patient plasma (bar = 100 μm, **arrowheads** indicate microvessels). The **(G)** number and **(H)** length of vascular sprouts (10 random viewfields) from AVSs cultured in indicated conditions. The results were from plasma samples of 26 patients collected at discharge and analyzed using 1-way analysis of variance (∗*P <* 0.05, ∗∗*P <* 0.01, ∗∗*P <* 0.001). **(I)** A cross-section view of a hematoxylin and eosin (HE)–stained AVS (**asterisk** indicates the vascular lumen, bar = 50 μm). The CD31^+^ microvessels of AVSs grown in **(J)** growth factor–rich medium (GRM) and **(K)** EV-supplemented GPM (bar = 25 μm). These are representative images from 22 AVSs reviewed by a mouse pathologist who was blind to the experimental conditions. Abbreviations as in Figure 1.

The results reported in Figure 1 led us to hypothesize that VWF^+^ pEVs carry angiogenic activity because increased permeability is considered to be an early stage of angiogenesis. When plasma was fractionated, we found that EVFP (Figure 2A) but not EVs (Figure 2D) from pre-LVAD samples induced significant angiogenesis of AVSs. In contrast, both the EVFP and EV fractions from post-LVAD plasma displayed proangiogenic activity (Figures 2B and 2E). The AVSs cultured with EVFP from post-LVAD samples developed long, mature vessels (Figure 2B) similar to those cultured with pre-LVAD samples (Figure 2A), whereas those cultured with EVs from post-LVAD samples developed dense, shorter, and hairlike vessels (Figure 2E). The VWF antibody that blocked endothelial permeability (Figure 1A) reduced the angiogenesis induced by EVFP but completely blocked EV-induced angiogenesis (Figures 2C and 2F). VWF (30 μL/mL) did not induce angiogenesis (Figures 2G and 2H). The CD31^+^ microvessels from AVSs cultured in GRM were larger and had intact vascular lumens (Figure 2J), whereas those in EV-supplemented GPM were often smaller, less intact, and sometimes occluded (Figure 2K). The EVs from LVAD patients contained 347.3 ± 111.3 pg/mL of vascular endothelial growth factor (VEGF), significantly higher than the 87 ± 26 pg/mL found in normal plasma (n = 26 for patients and n = 10 for control subjects; *t* test, *P <* 0.050).

**Figure 3:**
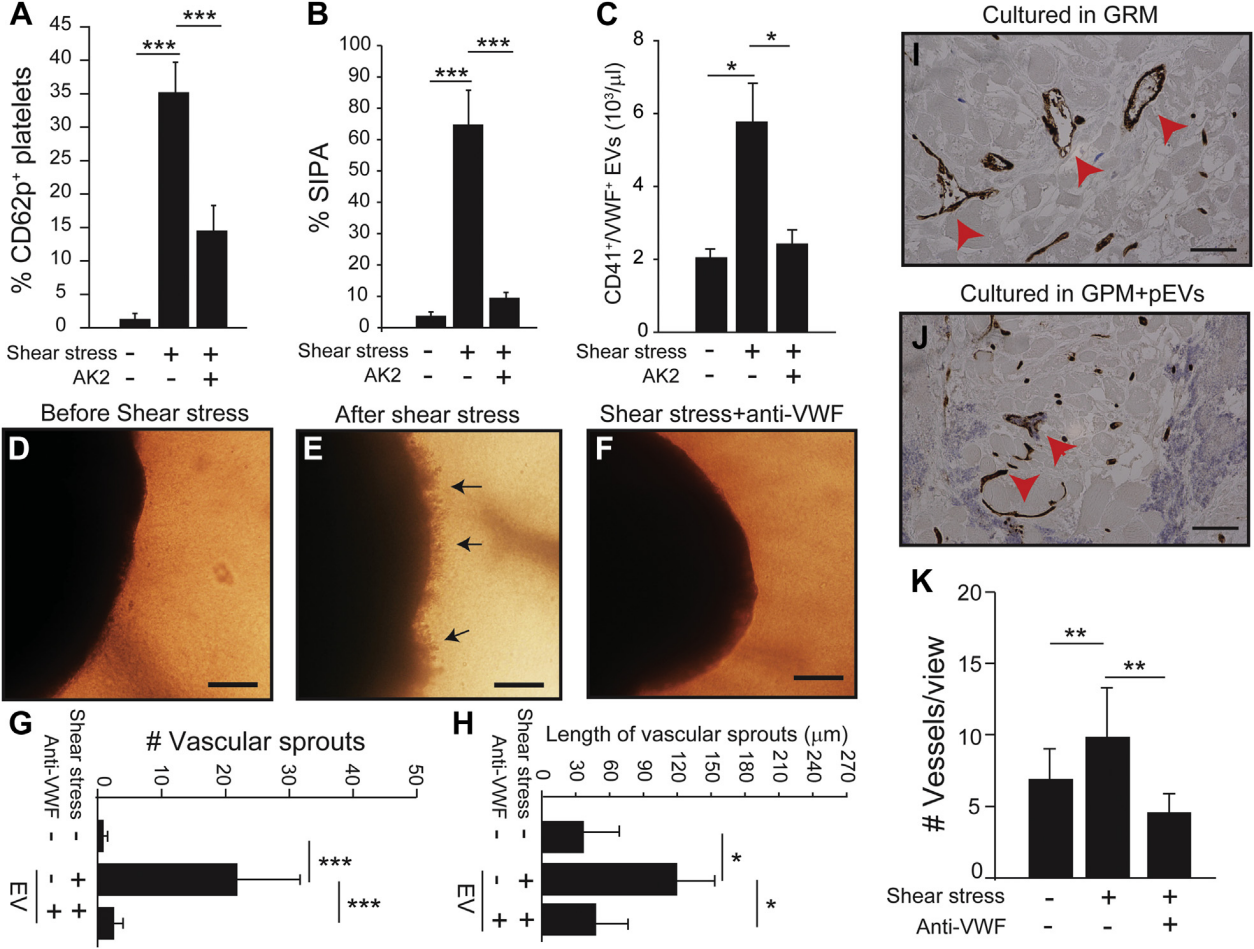
pEVs Promoted Angiogenesis in a VWF-Dependent Manner Normal platelets in platelet-rich plasma were **(A)** activated, **(B)** aggregated, and **(C)** produced EVs upon exposure to 110 dynes/cm^2^ of high shear stress for 5 minutes at 37 °C (n = 18 with the samples from each subject tested before and after shear exposure). **(D to F)** EVs from VWF-activated platelets (pEVs) (1 × 10^6^/μL) from shear-activated platelets but not from unsheared samples promoted angiogenesis from AVSs in GPM (bar = 100 μm, **arrow**: vascular sprouts). The **(G)** number and **(H)** length of vascular sprouts were quantified (n = 18). Representative images of CD31^+^ microvessels from AVSs cultured in **(I)** GRM and **(J)** pEV-supplemented GPM (bar = 10 μm) and **(K)** the summary of 9 independent experiments. The quantitative data were analyzed using 1-way analysis of variance or 1-way analysis of variance on ranks (∗*P <* 0.05, ∗∗*P <* 0.01, ∗∗*P <* 0.001). SIPA = shear-induced platelet aggregation; other abbreviations as in Figures 1 and 2.

To specifically examine the effects of pEVs, we exposed platelets from healthy subjects to 110 dynes/cm^2^ of HSS to mimic the condition of the LVAD-driven blood flow. Upon exposure, normal platelets expressed CD62p (Figure 3A), aggregated (Figure 3B), exposed PS (Supplemental Figure 2), and released pEVs (Figure 3C), which were reduced by the GP Ibα antibody AK2. This antibody blocks VWF binding to GP Ibα on platelets.^29^ We also detected shear-dependent hemolysis (Supplemental Figure 3). The pEVs from platelets exposed to HSS promoted angiogenesis in a VWF-dependent manner, but those from unsheared platelet-rich plasma did not (Figures 3D to 3F). The vascular sprouts from AVSs cultured in pEV-supplemented GPM were shorter, denser, and hairlike (Figure 3E), similar to those induced by EVs from LVAD patients (Figure 2). These pEVs contained 537.7 ± 36.6 pg/mL of VEGF (n = 6), significantly higher than that in the heterogeneous EVs from LVAD patients (n = 16; *t* test, *P =* 0.011). The CD31^+^ microvessels from AVSs cultured in pEV-supplemented GPM were small and often lacked intact vascular lumens (Figure 3I), whereas those in GRM were fully developed (Figure 3J). The number of CD31^+^ vessels was higher in AVSs cultured with pEV and was reduced by the VWF antibody (Figure 3K). For validation, we also used the 2-dimensional model of endothelial network formation assay to investigate the synergistic effects of pEVs and VWF on angiogenesis. Consistent with the data in Figure 3, pEVs (VWF^+^/CD41a^+^) promoted the formation of endothelial networks in a VWF-dependent manner, whereas purified VWF did not (Supplemental Figure 4). These data suggest that the blood from LVAD patients contained plasma-derived and EV-derived proangiogenic activities and that the latter promoted aberrant angiogenesis and was primarily derived from pEVs from the platelets activated by VWF under HSS.

### VWF was hyperadhesive in LVAD patients

**Figure 4:**
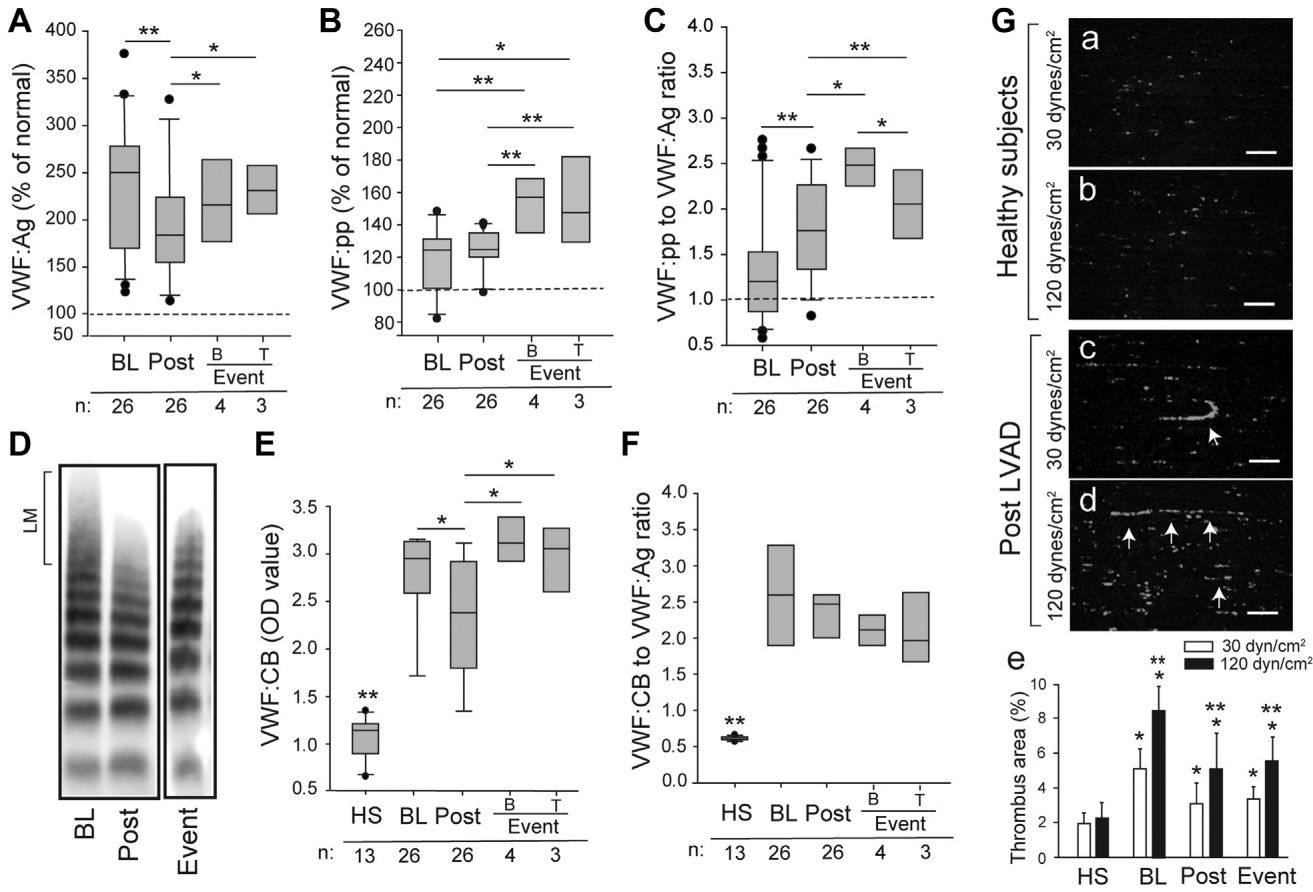
VWF in Patients Was Hyperadhesive **(A)** VWF antigen (VWF:Ag), **(B)** VWF propeptide (VWF:pp), and **(C)** the VWF:pp-to-VWF:Ag ratio measured at BL, at Post, and at clinical events of B or T (**dotted line** indicates levels from HS set as 100% or a ratio of 1). **(D)** VWF multimers in a patient at BL, Post, and clinical event (large VWF multimers [LM], with representative images from longitudinal samples from patients analyzed). **(E)** VWF binding to collagen (VWF:CB) and **(F)** its ratio to VWF:Ag of 13 HSs and 26 patients collected at BL, Post, and the clinical events of B and T. Sample sizes are marked at the bottom of the panel figures. The **arrows** in panels c and d refer to VWF formed string-like structures to which platelets bound. **(G) (a to d)** Representative images of platelet thrombi after 10 minutes of perfusing reconstituted blood under 30 and 120 dynes/cm^2^ of shear stresses (bar = 100 μm). **(e)** The areas covered by platelet thrombi were quantified for samples from 12 patients and 6 HS. The quantitative data were analyzed using either 1-way analysis of variance or 1-way analysis of variance on ranks (∗*P <* 0.05, ∗∗*P <* 0.01, ∗∗*P <* 0.001). For panel **e**, ∗*P <* 0.05 between 30 and 120 dynes/cm^2^ and ∗∗*P <* 0.01 vs HS. Abbreviations as in Figure 1.

VWF:Ag was significantly higher at baseline and was moderately reduced after LVAD; however, it remained higher than that of healthy control subjects (Figure 4A). VWF:Ag increased again in samples collected at the time of clinical events. VWF:pp was increased before and after LVAD implantation and rose further at clinical events (Figure 4B), leading to the significantly increased VWF:pp-to-VWF:Ag ratio (Figure 4C). While there was no difference in VWF:Ag and VWF:pp between patients who developed bleeding and those with thrombosis (Figures 4A and 4B), the VWF:pp-to-VWF:Ag ratio was greater in patients with bleeding than those with thrombosis (Figure 4C), suggesting a higher degree of endothelial activation in bleeding patients, consistent with the in vitro findings (Figures 1C and 1D). Large VWF multimers were lost in 92.3% (n = 24 of 26) of patients after LVAD implantation (Figure 4D), but their binding to collagen (VWF:CB), a common measure of VWF adhesive activity, was enhanced before (Figure 4E) and after adjustment for VWF:Ag (Figure 4F); however, no difference was detected between patients with bleeding and those with thrombosis. The plasma from LVAD patients also promoted a greater level of platelet thrombosis under an HSS of 120 dynes/cm^2^ than under an arterial shear stress of 30 dynes/cm^2^, with string-like structures formed under both levels of shear stress (Figure 4G). We also measured VWF cleaved by ADAMTS-13 and uncleaved VWF in EV and EV-free fractions of plasma samples collected at discharge of LVAD patients using mass spectrometry.^30^ The ratio of cleaved VWF in EVFP to that in EV fraction was 2.27 after adjustment for total VWF, suggesting that platelet- and pEV-bound VWF was significantly less cleaved. Together, these results suggest that: 1) the ECs underwent persistent exocytosis of VWF after LVAD implants and were subjected to additional stress at the time of severe bleeding or thrombosis; 2) plasma VWF in LVAD patients was hyperadhesive and capable of forming fibrillary structures under HSS.

### ADAMTS-13 activity was relatively low in LVAD patients

**Figure 5:**
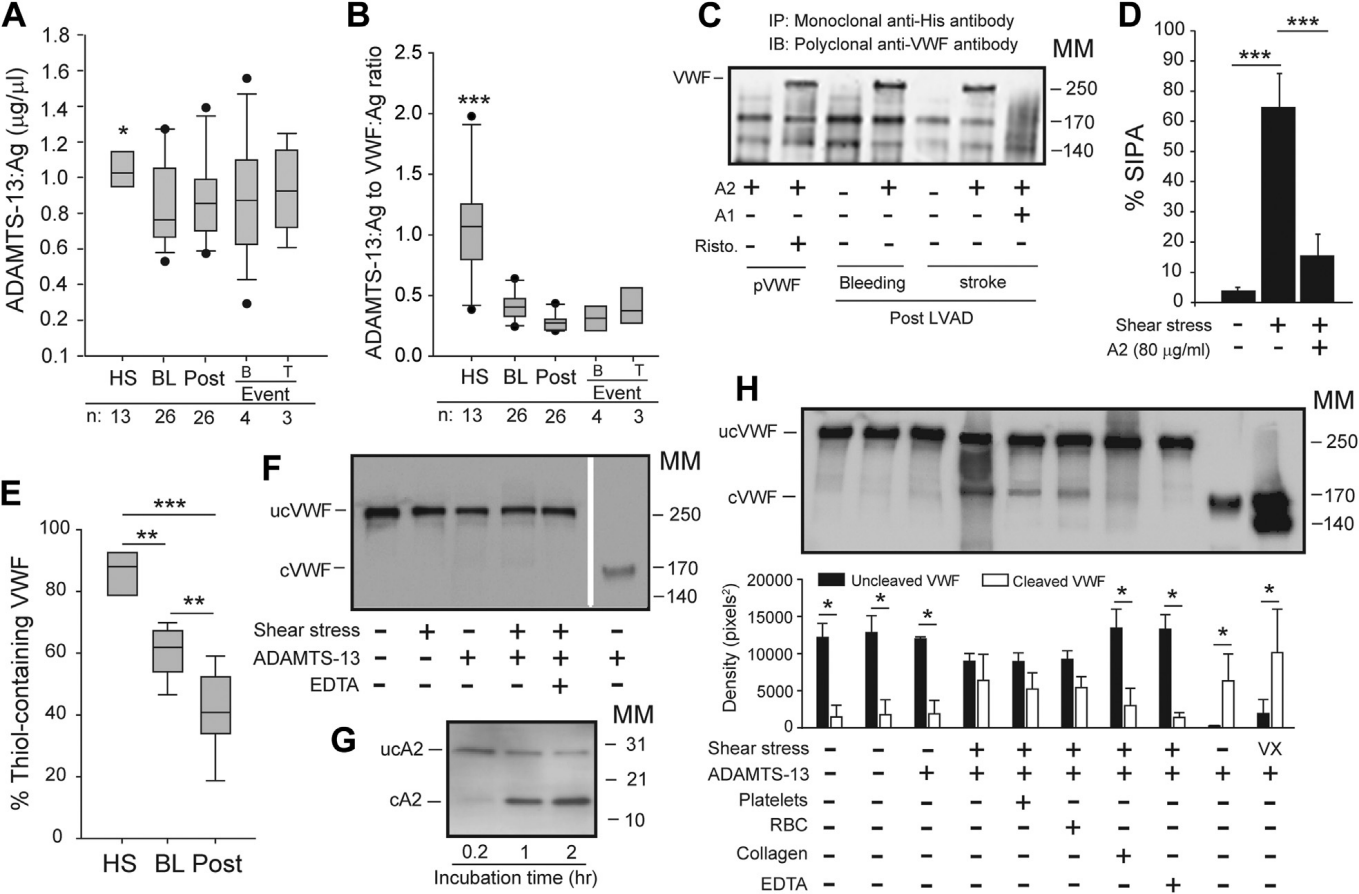
Shear-Induced VWF Activation and Cleavage **(A)** ADAMTS-13 antigen (ADAMTS-13:Ag) and **(B)** its ratio to VWF:Ag in samples from HS and patients sampled at BL, at Post, and at B or T events (sample sizes at the bottom of the panel figures, ∗*P <* 0.05 and ∗∗∗*P <* 0.001, HS vs other groups). **(C)** A2 precipitated VWF from the plasma of LVAD patients who developed bleeding or stroke post-LVAD and the precipitation was blocked by A1 (representative from 26 patients analyzed). A2 precipitated VWF purified from cryoprecipitate only after ristocetin (Risto.) treatment. **(D)** A2 also blocked SIPA of HS (n = 8/group). **(E)** Thiol-containing VWF precipitated from HS (n = 13) and patients at BL and Post (n = 26, ∗∗∗*P <* 0.001). **(F)** Cleaved VWF (cVWF) and uncleaved VWF (ucVWF) from HS exposed to 5 minutes of high shear stress (lanes 1-5) or in the static condition with 1.5 M urea and 1 mM of BaCl_2_ (lane 6). **(G)** A2 cleavage in the static condition. **(H) (Top)** cVWF and ucVWF from HS exposed to high shear stress for 60 minutes (lanes 1-8), under static incubation of 16 hours (lane 9), or exposed to a constant vortex for 60 minutes (lane 10). **(Bottom)** Densitometry of 8 independent experiments. All quantitative data were analyzed using 1-way analysis of variance. cA2 = cleaved A2; MM = molecular mark in kDa; RBC = red blood cells; ucA2 = uncleaved A2; ucVWF = uncleaved VWF; other abbreviations as in Figures 1 and 3.

The data in Figure 4 suggest that VWF lost large multimers in plasma because it had enhanced binding to platelets (gain of function [GOF]). We conducted 2 sets of experiments to dissect the underlying mechanism of this GOF phenotype. First, we found that ADAMTS-13:Ag was moderately reduced in LVAD patients (Figure 5A) as compared with the significantly increased VWF:Ag (Figure 4A), leading to a drastically reduced ADAMTS-13:Ag-to-VWF:Ag ratio at comparable levels between patients with severe bleeding and those with thrombosis (Figure 5B). However, ADAMTS-13 in patients’ plasma cleaved the exogenous VWF peptide at a rate comparable to that of normal plasma (Supplemental Figure 5). The data presented in this section and early sections collectively suggest that ADAMTS-13 found in LVAD patients remained active and did not cleavage VWF in patients' blood because kinetic deficiency caused by a significantly reduced ADAMTS13:Ag-to-VWF:Ag ratio. The finding also raised the possibility of other pathways contributing to VWF hyperadhesive activity.

### HSS activated VWF by inducing conformational changes

Second, we examined the structural changes of VWF in LVAD patients using several techniques. First, the co-immunoprecipitation assay showed that a recombinant A2 protein bound to the exposed A1 domain^28^ to form a complex with VWF in the plasma samples of LVAD patients (Figure 5C). In contrast, A2 bound VWF from healthy subjects only in the presence of ristocetin (Figure 5C), which activates VWF to bind its platelet receptor.^29^ Second, A2 blocked SIPA (Figure 5D), which is induced by the binding of shear stress–activated VWF to platelets.^25^ Third, the thiol-containing VWF accounted for 86.4% ± 7.1% in healthy subjects but was reduced to 61.0% ± 8.1% in patients at baseline and decreased further to 42.3% ± 13.0% after LVAD implantation (Figure 5E), with a parallel increase of VWF in the supernatant (Supplemental Figure 6). These results suggest that VWF multimers in LVAD patients were oxidized and underwent conformational changes to expose the A1 domain.

Exposing normal platelet-rich plasma to HSS for 5 minutes failed to induce VWF cleavage (Figure 5F), but it did induce significant platelet activation and aggregation by VWF (Figures 3A to 3C). As a control, VWF was cleaved in static conditions after incubation for 16 hours in the presence of 1.5 M urea and 1 mM of BaCl_2_. An isolated A2 required 1 hour to be cleaved without added chemicals (Figure 5G). In contrast, VWF was partially cleaved after exposure to HSS for 60 minutes (Figure 5H). The cleavage was not affected by platelets (2 × 10^5^/μL) or erythrocytes (2 × 10^6^/μL), but it was prevented by collagen (10 μg/mL). The cleavage was similarly induced under a turbulent flow generated in a vortex for 60 minutes.^31^ These results suggest that VWF binding to platelets preceded VWF cleavage by ADAMTS-13 under HSS.

### Shear stress enhanced VWF-mediated hemostasis in mice

**Figure 6:**
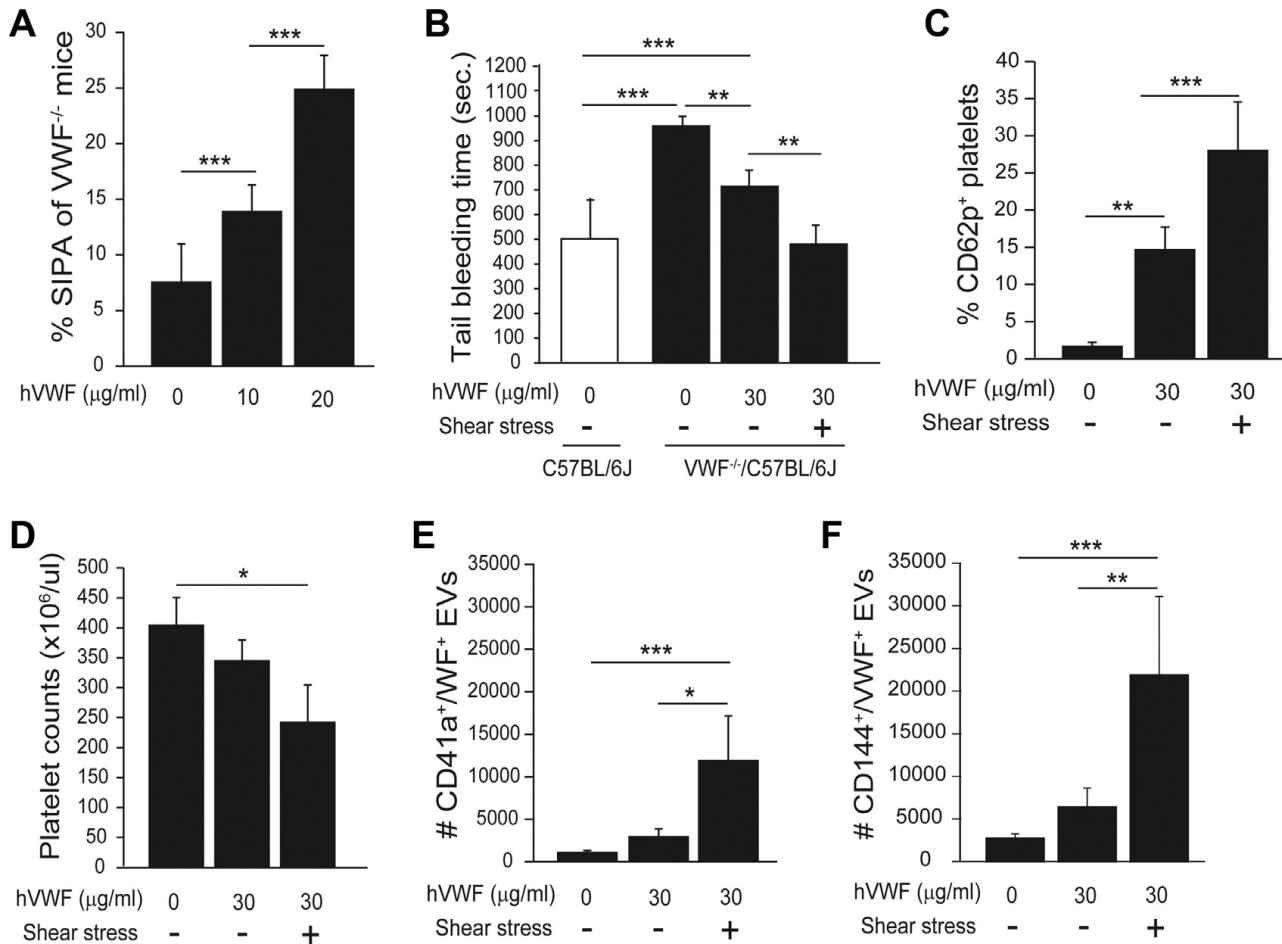
Shear-Activated VWF Rescued Hemostasis of VWF^-/-^ Mice **(A)** Human VWF (hVWF) aggregated mouse platelets under high shear stress (n = 6). **(B)** Tail bleeding measured 30 minutes after mice were infused with VWF before and after exposure to high shear stress (n = 9 in each group). Blood samples were collected from VWF^-/-^ mice 30 minutes after VWF infusion and analyzed for **(C)** CD62p expression, **(D)** platelet counts, **(E)** pEVs, and **(F)** endothelial EVs. All data were analyzed using 1-way analysis of variance (∗*P <* 0.05, ∗∗*P <* 0.01, ∗∗∗*P <* 0.001). Abbreviations as in Figures 1 and 3.

Because there is no mouse model of LVAD, we tested the effects of HSS on VWF in VWF-deficient mice. This experiment was possible because: 1) the HSS induced mouse SIPA (Supplemental Figure 7) and promoted human VWF to aggregate mouse platelets (Figure 6A); and 2) we have shown that VWF maintains its shear-induced active conformation for more than 5 hours after shear exposure has stopped,^32^ allowing sufficient time for experiments to be conducted. Hemostasis was restored partially in VWF^-/-^ mice infused with VWF and completely with the VWF that was exposed to HSS for 5 minutes at 37 °C (Figure 6B, Supplemental Figure 8). Platelets from VWF^-/-^ mice infused with sheared VWF expressed CD62p (Figure 6C), developed moderate thrombocytopenia (Figure 6D) and generated more VWF^+^ pEVs (Figure 6E). These mice also had elevated levels of endothelial EVs (Figure 6F). These results suggest that VWF exposed to HSS fully restored the hemostasis of VWF^-/-^ mice.

## Discussion

**Figure 7:**
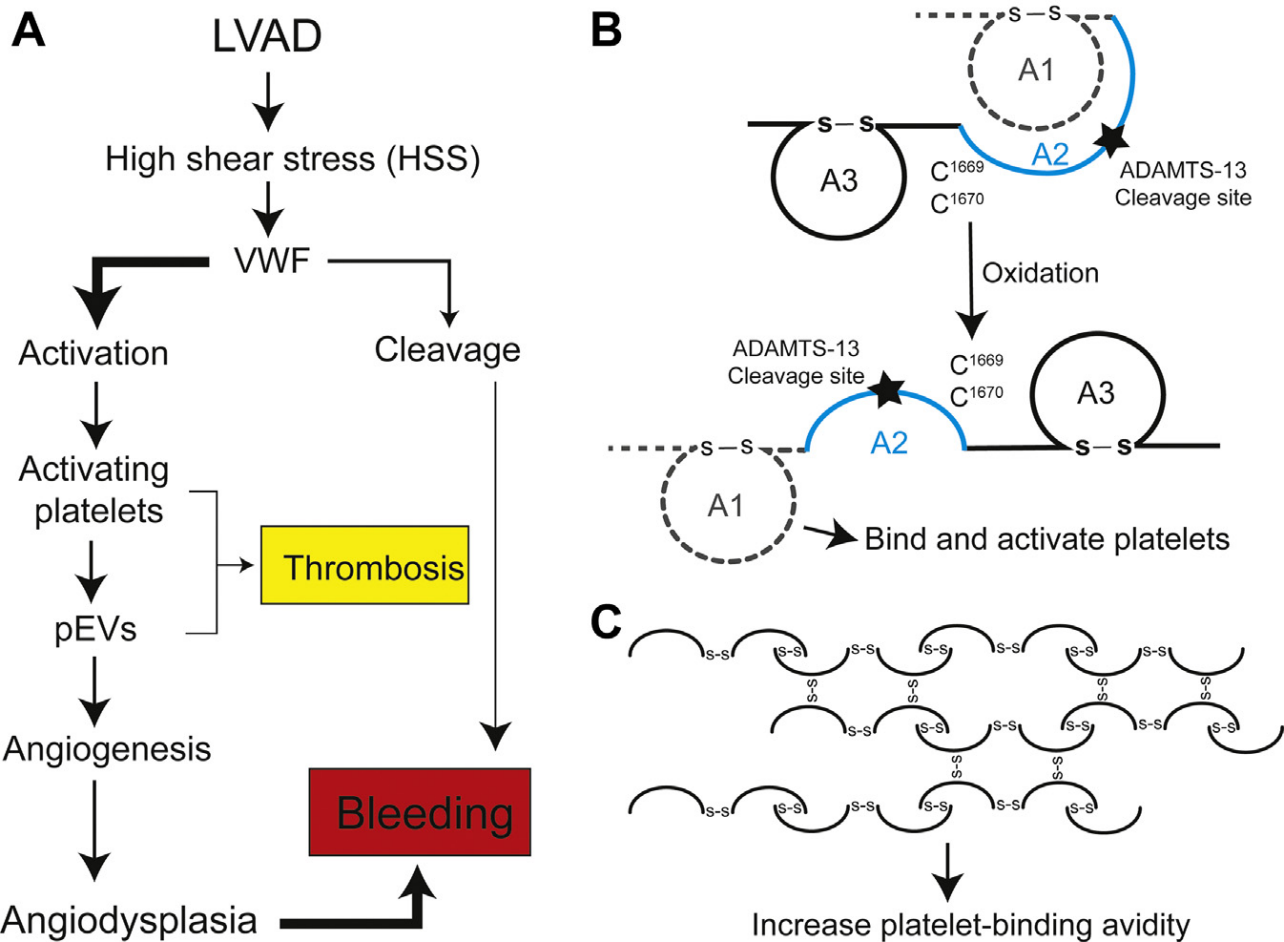
Schematic Illustration of Structural and Functional Changes of VWF in an LVAD-Driven Blood Flow **(A)** High shear stress (HSS) activates VWF (gain of function) and facilitates VWF cleavage by ADAMTS-13 (loss of function), with the former being the predominant pathway in patients on LVAD supports. **(B)** The 2 vicinal cysteines in the A2 domain can be oxidized to disrupt the A1-A2 complex so that the A1 exposed (activated) VWF can form a complex with isolated A2. **(C)** Cysteine thiols of VWF can be oxidized to form intermultimeric disulfide bonds under HSS and thus cannot be precipitated by the active thiol beads, which form mix disulfide bonds with surface exposed free thiols on VWF.^68^ Abbreviations as in Figures 1 and 3.

In contrast to the widely held belief, our data demonstrate that VWF in LVAD patients undergo conformational changes to bind and activate platelets and to produce VEGF-carrying pEVs that promote aberrant angiogenesis measured in both 2- and 3-dimensional angiogenesis assays (Figure 7A, Supplemental Figure 4). While this study was conducted on samples from patients supported by axial-flow LVADs, the results are applicable to those on centrifugal LVADs because differential VWF characteristics remain poorly defined between the 2 types of LVADs. For example, centrifugal LVADs tend to have lower pump speed than axial-flow LVADs^33^^,^^34^; some studies report similar VWF characteristics between the 2 types of LVADs,^33^^,^^35^ whereas others show reduced VWF “fragmentation” with centrifugal LVADs without providing evidence that the fragmentation is caused by VWF cleavage and changes VWF adhesive activity.^36^^,^^37^ Furthermore, the laboratory assays commonly used to study VWF in LVAD patients (eg, multimers and collagen binding) are limited and insufficient to detect the structural changes of VWF under HSS, especially related to platelet and endothelial functions. We employed multiple tests to examine the structure, conformation, oxidation, and platelet activating activity of VWF before and after LVAD implantation. We made several novel observations.

First, the EVs from LVAD patients induced significant vascular permeability and aberrant angiogenesis in a VWF-dependent fashion (Figures 1 and 2). This finding is consistent with previous reports that EVs can transmigrate through the endothelial barrier^38^^,^^39^ and that VWF plays a role in the process.^28^^,^^40^ VWF did not promote angiogenesis directly, but likely served as a coupling factor that tethered pEVs to ECs in flowing blood, likely through simultaneous binding to GP Ibα on pEVs and α_v_β_3_ integrin and CD62p on ECs^41^^,^^42^ to locally concentrate VEGF for angiogenesis.^43^ VEGF is stored in the α-granules of platelets, which releases it upon activation.^44^ VEGF induces VWF release from ECs,^45^ causes endothelial permeability, and promotes the formation of immature and “leaky” vessels.^46^ VEGF is also carried by EVs in patients with diabetes mellitus.^47^ In addition, EVs from endothelial cells contain angiopoietin-like protein 2,^48^ which also promotes angiogenesis. Our finding is supported by previous reports that EVs deriving from adipocytes and leukocytes promote angiogenesis^49^^,^^50^ through distinct but closely related pathways.^50^, ^51^, ^52^, ^53^ It is also interesting to note that EV-derived proangiogenic activity was enhanced in a swine model of obesity and hyperlipidemia,^54^ which are key causal factors for the coronary heart disease that could lead to heart failure. EVs can also carry molecules that may be inhibitory to angiogenesis, such as miR-24,^55^ which inhibits cell proliferation.

Second, we show that the VWF in LVAD patients was hyperadhesive, activating platelets (Figure 1) and enhancing VWF:CB under static and flow conditions (Figure 4). VWF:CB has been previously reported to both increase^56^^,^^57^ and decrease in LVAD patients.^35^^,^^58^^,^^59^ However, reports on the latter compare VWF:CB before and after LVAD. This comparison may obscure the true level of VWF adhesive activity because patients with end-stage heart failure have elevated levels of VWF in comparison with healthy subjects,^60^, ^61^, ^62^ leading to the impression that VWF:CB is reduced in LVAD patients. We found that VWF:CB was moderately reduced post-LVAD, but it remained higher than that of healthy subjects.

The finding that VWF lost large multimers but remained hyperadhesive, albeit at reduced levels (Figure 4), defines a GOF phenotype with large VWF multimers lost to enhanced binding to platelets, consistent with increased VWF on platelets and pEVs found in the post-LVAD samples (Figure 1). Consistent with the notion, VWF found on pEVs was significantly less cleaved than VWF found in plasma, suggesting that platelets were activated to generate pEVs by less cleaved and thus more adhesive VWF, which also mediated EV-EC interaction to promote angiogenesis. This GOF phenotype is supported by the finding that the VWF exposed to HSS was more effective in restoring hemostasis in VWF-null mice (Figure 6), not reducing it, as one would expect if HSS reduces the adhesive activity of VWF by inducing excessive cleavage. This GOF phenotype resembles type 2B von Willebrand disease, in which large hyperadhesive VWF multimers bind to platelets, thus selectively being removed from plasma.^63^ Furthermore, this GOF phenotype suggests that defining excessive cleavage by the loss of large VWF multimers in plasma may be inaccurate because large and hyperadhesive VWF multimers bind to platelets and are thus selectively and disproportionally removed from plasma, artificially increasing the amount of cleaved soluble VWF.

Third, we provided several lines of evidence to show that VWF was activated in LVAD patients through structural changes. The first is that the A1 and A2 domains form a complex in plasma VWF during homeostasis^64^ to prevent spontaneous platelet binding and cleavage. This A1-A2 complex is formed when the vicinal cysteines C^1669^ and C^1670^ in the A2 domain are reduced and is disassociated when they are oxidized.^65^ We show that the A1 and A2 complexes are disassociated to expose the A1 domain of VWF in LVAD patients (Figure 5C), as schematically illustrated in Figure 7B, but did not induce cleavage likely because the VWF was oxidized (Figure 5E), making it resistant to ADAMTS-13^66^^,^^67^ and forming laterally associated hyperadhesive fibrils,^68^ as schematically illustrated in Figure 7C. This oxidative blockage of VWF cleavage^30^ is accelerated by HSS^66^ and VWF-bound hemoglobin,^69^ and the latter is a potent oxidant. The second is the kinetic ADAMTS-13 deficiency that developed in LVAD patients because of drastically reduced ADAMTS-13:Ag-to-VWF:Ag ratio (Figure 5B). These results from patients are further supported by in vitro experiments showing that the HSS-induced VWF binding to platelets was significantly faster than the HSS-induced cleavage by ADAMTS-13 (Figures 3A to 3C and 5F to 5H).

### Study limitations

This study is limited by its small sample size of patients, which did not allow sufficient stratification to conclusively link VWF hyperadhesive activity to the clinical outcomes of LVAD patients. However, defining VWF phenotypes in LVAD patients and understanding their underlying causes would allow us to identify key markers linked to VWF reactivity and to study the causal relationship between the aVWS and hemostatic complications of LVAD patients. The patient recruitment is ongoing, with the follow-up period extended to 24 months, including multiple time points of clinical assessments and blood sampling. The ongoing recruitment will allow a sufficient sample size for developing a composite score system that combines VWF and other laboratory and clinical measurements to assess the bleeding risk of patients. Second, the patient study was conducted with blood samples collected from patients on axial-flow LVADs, whereas most new patients receive centrifugal LVADs. However, our ongoing recruitment will allow us to collect samples from patients on both types of LVADs in order to compare VWF profiles so that we will be able to more precisely define the impact of shear stress on VWF. Third, because of a limited plasma volume, we were unable to map the specific amino acids involved in forming intermultimer disulfide bonds or being oxidized. We are developing a new mass spectrometric protocol to overcome this technical difficulty. Finally, because of the lack of suitable research on LVADs in mouse models, we were unable to test our hypothesis of LVAD-induced GOF VWF and its synergistic actions with pEVs to promote angiogenesis in vivo. We can overcome this obstacle in experiments on large animals in the future.

## Conclusions

In summary, we demonstrate that VWF was not excessively cleaved but became hyperadhesive in the majority of LVAD patients. We identified platelet microvesiculation, resulting from the LVAD-driven HSS blood flow, as a key contributor to the hemostatic dysfunction, endotheliopathy, and aberrant angiogenesis found in LVAD patients. These findings could have a direct impact on the clinical management of patients on LVAD support. For example, aspirin, which is commonly prescribed after LVAD, may not be effective in preventing this VWF-mediated platelet activation and EV-driven angiogenesis because shear-induced platelet activation is insensitive to aspirin.^70^^,^^71^ Another finding that requires further investigation is that all patients received dipyridamole post-LVAD, which is a phosphodiesterase inhibitor that reduces platelet reactivity and vascular tension by blocking the adenosine metabolism by erythrocytes and vascular endothelial cells. A recent study found that the phosphodiesterase 3A inhibitor reduced platelet microvesiculation,^72^ but dipyridamole failed to reduce plasma levels of pEVs in the present study. These findings are also significant for understanding the complications associated with other medical devices that significantly alter blood hydrodynamics (eg, percutaneous microaxial LVAD and extracorporeal membrane oxygenation).

Perspectives **COMPETENCY IN MEDICAL KNOWLEDGE:** VWF is an adhesive ligand critical for hemostasis, but it is also an acute phase reactant involved in inflammation, angiogenesis, and thrombosis. These diverse activities derive from the size and structure of VWF multimers. How hydrodynamic changes induced by LVAD-driven blood flow alter VWF structure remains poorly understood. This study shows that VWF is predominantly activated by HSS and oxidative stress, calling for more comprehensive study of VWF in the pathogenesis of LVAD-induced bleeding complications.

**TRANSLATIONAL OUTLOOK:** The current study used extensive technologies to define the structure-and-function correlation of VWF multimers found in LVAD-driven blood flow. The study raises the question as whether VWF multimers are excessively cleaved by the metalloprotease ADAMTS-13 or activated to bind platelets, and more importantly, how the 2 opposing processes reach the equilibrium in patients on LVAD supports. This study identified shear-induced and VWF-mediated platelet microvesiculation as a potential key contributor to angiodysplasia and associated bleeding in patients on LVAD supports. The clinical and laboratory VWF variables obtained from this study could allow us to develop a composite score that predicts the bleeding risk and need for targeted prophylaxis of individual patients based on their VWF profiles.

## Funding Support and Author Disclosures

The authors have reported that they have no relationships relevant to the contents of this paper to disclose.
