# Supplementary material for: Hyperadhesive von Willebrand Factor Promotes Extracellular Vesicle-Induced Angiogenesis: Implication for LVAD-Induced Bleeding
Source: JACC Basic Transl Sci. 2022 Mar 28;7(3):247–61. doi: 10.1016/j.jacbts.2021.12.005 (PMC8993768; doi:10.1016/j.jacbts.2021.12.005)
Supplement: Supplemental Data [file mmc1.docx]

**Supplemental APPENDIX**

**Sample tests and result validation**

We took several quality control steps to ensure the reliability and reproducibility of the data, as we previously discussed for large population studies (1,2). To ensure reproducibility, we randomly selected 10% plasma samples from patients and divided each into 7 aliquots. These sample aliquots were tested daily for 7 days to generate coefficient of variance (CV) of a given test. The CVs will allow us to define variations found in individual experiments and those associated with a specific assay. In addition, samples with known values (calibration standard) were included in each assay to validate the fidelity of the equipment. Each sample was analyzed in triplicates in all assays to ensure the consistency of measurements.

Samples sent for analyses by outside vendors (e.g., ADAMTS-13 activity) were coded so that the technical staff was blinded to the condition of samples. For tests performed in the laboratory at the Bloodworks Research Institute, all samples were coded upon collection. The technical staff and fellows who performed the tests did know the conditions of samples when performing these tests. All results were analyzed by a biostatistician who was blinded to the experiments.

**Endothelial network formation assay**

Platelet EVs were generated from platelet-rich plasma stimulated with 25µg/ml of ultra-large VWF multimers (ULVWF), which are intrinsically hyperadhesive and capable of activating platelets without induction (3,4), and 20 µM of ADP. ULVWF multimers were collected from the supernatant of primary EC cultures stimulated with 25 µM of histamine (10 min at 37^o^C), as we previously described (3). The numbers of pEVs from VWF-activated platelets (VWF^+^/CD41a^+^) were quantified using flow cytometry (5) and tested for promoting angiogenesis using the network formation assay.

The two dimensional network formation assay was modified from a method reported previously (6). Briefly, wells from 48-well plates were coated with 100 µl of Matrigel dissolved at -4^o^C. The plates were then warmed to 37^o^C for 60 min to allow the matrix to form. Primary ECs were plated in these wells at the density of 1x10^6^/ml (100 µl/well) and treated with the medium (100 µl/well) containing testing agents, as specified in the Results section. The VWF^+^/CD41a^+^ pEVs were tested at a final density of 2x10^3^/µl. The cells were cultured at 37^o^C for 6 hrs and were then scanned for network formation, which was quantified as the numbers of endothelial lumen under review fields at 100x magnifications. The control cells were cultured in the basal medium with and without 10 µg/ml VEGF (Cat #: Ab9571, Abcam, Waltham, MA)

**Plasma fractionation**

EVs and EV-free plasma (EVFP) were prepared, as we previously described (5). Briefly, plasma from patients and control subjects (0.38% sodium citrate as anticoagulant, final concentration) was centrifuged at 13,000xg for 10 min at 4^o^C to collect cell-free plasma, which was then centrifuged at 100,000xg for 60 min (twice) at 4^o^C to collect EV pellets, which were suspended in DMEM medium (ThermoFisher Scientific). The supernatant from two sequential ultra-centrifugations was tested as EVFP. EVs and EVFP were tested immediately after preparation.

**Flow cytometry**

Plasma (20 µl) was incubated with PE-conjugated annexin V (Cat #: ALX-209-251-T100, Enzo life science, Farmingdale, NY) in 80 µl of calcium-containing annexin V binding buffer (eBioscience, San Diego, CA), together with each of the following antibodies: FITC-anti-VWF (Cat #: ab8822, Abcam, Cambridge, UK), APC-anti-CD41a (Cat #: 17-0419-42, Life Technologies, Carlsbad, CA), and APC-anti-CD144 (Cat #: 17-1441-82, eBioscience, San Diego, CA) for 30 min at room temperature. To specifically detect EVs from VWF-activated platelets (pEVs), plasma was incubated with the VWF and CD41a antibodies. The plasma was diluted to 500 µl with PBS containing 1% paraformaldehyde and analyzed using flow cytometry (Becton Dickinson, San Jose, CA) (7,8). EVs were identified first by their size (<1.0μm) using megamix microbeads of 0.3, 0.6, and 1.2 µm and then by the cell markers. Flow cytometry was also used to detect platelet activation using a PE-CD62p antibody (Cat #: 555524, BD biosciences, San Jose, CA) and EV-induced EC activation by measuring levels of CD144^+^/annexin V^+^ endothelial EVs (eEVs). VWF-bound eEVs were detected by the VWF and CD144 antibodies.

**Cone-and-plate viscometer assay**

Shear-induced platelet aggregation (SIPA) was induced on a cone-and-plate viscometer (ThermoFisher Scientific, Waltham, MA) using a 1/3 degree cone and set at a constant rate, as we previously described (9,10). This assay measures the rate of platelet aggregation induced by VWF activated by high shear stress. Briefly, blood samples were collected from healthy subjects (0.38% final concentration of sodium citrate as anticoagulant) and centrifuged at 150xg for 20 min at 24^o^C to obtain platelet-rich plasma (PRP, viscosity ~ 1 cp). The PRP (500 µl) was loaded onto the cone-and-plate viscometer and exposed to rotational shear stress of 110 dynes/cm^2^ for times indicated in individual experiments. For this study, 110 dynes/cm^2^ of shear stress was defined as high shear stress (HSS) because it is commonly found in LVAD-driven blood flow. After exposure to shear stress, PRP was analyzed for shear-induced platelet activation detected by a PE-CD62p antibody (Cat #: 555524, BD biosciences) and microvesiculation detected by an anti-CD41a antibody (Cat #: 11-0419-42, Affymetrix, San Diego, CA) at the preset EV gate using flow cytometry (BD Bioscience).

SIPA was quantified by the reduction of single platelet counts measured in a Coulter counter (Coulter Electronics, Miami, FL) after shear exposure (11). Platelet counts in unsheared PRP served as the baseline for SIPA calculation. The antibody AK2 (Cat #: MA5-16565, Invitrogen, , San Diego, CA), which binds the GP Ibα subunit of the GP Ib-IX-V complex on platelets to block VWF binding (12), or A2 protein, which binds the VWF A1 domain (8), were tested for blocking the shear-induced VWF-platelet interaction. In some experiments, human VWF multimers purified from plasma cryoprecipitate (11) were exposed to high shear stress, as indicated in individual data sets.

**Detection of EV-bound vascular endothelial growth factor (VEGF)**

EVs from plasma were purified using the ultracentrifugation method, as we previously reported (8,13). In brief, 1 ml of platelet-poor plasma (PPP) from patients was centrifuged at 1,600xg for 10 min at room temperature to remove cells and platelets. The cell-free supernatant was centrifuged at 13,000xg for 5 min at 4^o^C to remove large cellular fragments and then centrifuged at 100,000xg at 4​^o^C for 60 min (twice) to collect EV pellets, which were solubilized in 1% SDS lysis buffer and analyzed for VEGF using ELISA, according the manufacturer’s instructions (Cat #: Ab233625, Abcam). To specifically measure VEGF in platelet EVs, platelet-rich plasma (PRP) collected from healthy subjects (0.38% sodium citrate as anticoagulant) was first subjected to the pathological high shear stress of 110 dynes/cm^2^ for 10 min at 37^o^C. The PRP was centrifuged at 1,600xg for 10 min at room temperature to collect the supernatant as cell-free plasma, which was centrifuged at 13,000xg for 5 min at 4^o^C and then at two sequential 100,000xg centrifugations, each for 60 min at 4^o^C, to collect pEVs for VEGF measurements. Normal plasma contains 0–115 pg/ml of VEGF, but its level is significantly higher in serum at 62–707 pg/ml (14,15), suggesting a substantial release of VEGF from activated platelets during clot formation.

**Detection of thiol-exposed VWF**

We have previously shown that plasma VWF multimers contain free thiols on their surface that can be oxidized to form intermultimer disulfide bonds (11) to form hyperadhesive fibrils under high shear stress (16). Using this technology, we measured the thiol-disulfide-bond state of VWF multimers found in patients before and after LVAD implants. In brief, plasma samples from patients collected before and after LVAD implantation were incubated with 100 µM of maleimide-PEO_2_-biotin (ThermoFisher Scientific) for 30 min at room temperature and then treated with 200 µM glutathione (GSH, Sigma Aldrich) to stop the reaction. The plasma was then incubated with sepharose-streptavidin beads (Sigma Aldrich, St. Louis, MO) for 30 min at room temperature and centrifuged at 13,000xg at 4℃ for 10 min. The beads were washed with PBS and the precipitated proteins were released from the beads using the SDS-elution buffer. VWF captured by the maleimide-beads and that remained in the supernatant were measured separately using a commercial ELISA kit (Cat #: ab223864, Abcam). The thiol-containing VWF multimers (precipitated by maleimide-PEO2-biotin) are in the reduced state because they formed mixed covalent bonds with maleimide and thus were precipitated. In contrast, oxidized VWF multimers remained in the supernatant after maleimide precipitation because their free thiols are oxidized to form disulfide bonds.

**Collagen binding assay for VWF (VWF:CB)**

VWF:CB was measured using a commercial collagen binding assay kit (Sigma Aldrich), as we previously described (8). Briefly, 96-well plates were coated with Type III collagen overnight at 4°C and blocked with 2.5% bovine serum albumin (BSA) for 2 hrs at room temperature. Plasma samples from patients and controls were diluted (1/10) and incubated with the collagen coated plates for 2 hrs at 37°C. After washing, the plates were incubated with the HRP-conjugated polyclonal VWF antibody (Cat #: P022602-8, Agilent, Santa Clara, CA) for 2 hrs at room temperature. At the end of incubation, the plates were washed again with PBS to remove unbound antibodies, TMB was added and the samples were analyzed at OD450 nm in a plate reader (Molecular Device, Sunnyvale, CA).

**Detection of EV-induced activation of endothelial cells**

Human umbilical cord endothelial cells (HUVECs; ATCC, Manassas, VA) were grown to 80% confluence in the endothelial growth medium (Sigma Aldrich, St. Louis, MO) and then detached using 2% EDTA. After washing with PBS, the cells (20 µl at 1x10^6^/ml) were incubated with 10 µl of plasma from patients or healthy subjects for 30 min at 37℃ on a desktop shaker (25µM histamine [Sigma Aldrich] as positive control). After the incubation, cells were washed with warm PBS and incubated with FITC-CD144 antibody (Cat #: 560874, BD biosciences, Franklin Lakes, NJ) and APC-annexin V (BMS306APC-100, eBioscience, San Diego, CA) for 30 min at room temperature and analyzed using flow cytometry. The exposure of anionic phospholipids detected by annexin V was used as the marker of EC activation.

**Vascular permeability assay**

HUVECs were cultured on collagen-coated Transwells (0.4 µm pore, Corning) in endothelial cell growth medium (Sigma Aldrich) until reaching confluence. They were then incubated with plasma from LVAD patients or healthy donors for 3 hrs at 37°C in the presence or absence of the VWF-blocking antibody. After washing with PBS, cells were incubated with FITC-dextran (70 kDa, 100µg/ml, Sigma Aldrich) for 30 min at 37^o^C. The medium in the bottom chambers were analyzed for FITC-dextran fluorescence using a plate reader (Molecular Device).(8)

**Detection of VWF cleavage by ADAMTS-13**

We used two methods to measure the cleavage of VWF by ADAMTS-13. First, we have previously generated a goat antibody (A2-1606) against a synthetic VWF peptide C-terminal to the cleavage site (m^1606^vtgnpasdeIkrl, Bethyl Laboratories, Inc. Montgomery, TX) and used this antibody to specifically detect VWF cleaved by ADAMTS-13 (10). For this method, plasma samples (1/20 dilution) from patients and healthy controls were separated by 4% SDS-polyacrylamide gel electrophoresis (PAGE) under reduced conditions and transferred to PVDF membrane (Bio-Rad, Hercules, CA). After blocking non-specific binding by T20 blocking buffer (ThermoFisher Scientific, Waltham, MA) for 30 min at room temperature, the membrane was incubated with an HRP-conjugated rabbit anti-human VWF antibody (Cat #: P022602-8, Agilent, Santa Clara, CA) that recognizes primarily uncleaved VWF overnight at 4°C, followed by ECL™ Western Blotting Detection Reagent (GE Healthcare, Piscataway, NJ). The membrane was then stripped with the Restore™ Plus Western Blot Stripping Buffer (Thermo Scientific), blocked with BSA, and incubated overnight at 4^o^C with the antibody A2-1606. After washing, the bound antibody was detected by an HRP-conjugated rabbit anti-goat IgG (Invitrogen, A16136, San Diego, CA) (60 min at room temperature). The kinetics of VWF cleavage by ADAMTS-13 was quantified by the density of uncleaved and cleaved VWF bands using ImageQuant TL. The second is a commercial assay that measures the cleavage of a 73 amino acid peptide from the VWF A2 domain (FRET-73, Technoclone Inc., Vienna, AUSTRIA) by ADAMTS-13 in plasma of patients and controls. These two assays measured the cleavage of VWF and VWF-activity of ADAMTS-13 found in patient plasma, respectively.

**Immunoprecipitation**

A recombinant A2 protein (G^1481^–R^1668^) was generated in *E. coli* as a fusion protein with an N-terminal 6×His-tag and used to precipitate A1 exposed VWF multimers, as we have previously described (8). Briefly, plasma samples from LVAD patients (50 µl) were incubated with 80 µg/ml of A2 for 30 min at room temperature followed by incubation with the anti-His antibody (Cat #: ab1187, Abcam) that was coupled with Sepharose-conjugated protein A/G beads (ThermoFisher Scientific) overnight 4°C on a parallel shaker. They were then centrifuged at 13,000xg for 20 min at 4^o^C to precipitate the A2-bound VWF, which was decoupled from beads, separated through 4% SDS-PAGE, and detected with a HRP-conjugated polyclonal VWF antibody (Cat #: P022602-8, Agilent) using immunoblot. Plasma from healthy subjects treated with ristocetin (10 mg/ml; Helena Laboratories), which activates VWF, before A2 precipitation and patient plasma treated with A2 together with the VWF A1 domain (400µg/ml, D^1261^-T^1468^; U-Protein Express BV, Utrecht, Netherlands) served as controls.

**Flow Chamber assay**

We measured platelet adhesion and thrombus formation on the collagen matrix using a microfluidic chamber system (Cellix Fluoro^+^, Cellix, Dublin, Ireland), as we previously reported with minor modifications (17,18). Briefly, microfluidic channels were incubated with type I fibrillary collagen (5µg/ml, Helena Laboratory, Beaumont, TX) overnight at 4^o^C, washed with PBS, and then perfused with reconstituted blood at a flow rate that generated shear stresses of either 30 or 120 dynes/cm^2^ for 5 min at room temperature. The channels were washed with PBS and observed under an inverted stage microscope (Olympus, IX81) with images taken from 10 randomly selected view-fields. The formation of platelet thrombi on the collagen matrix was quantified as the area covered by thrombi using NIH Image J software.

To prepare reconstituted blood, whole blood was collected from healthy subjects using ACD as anticoagulant. The blood was centrifuged at 150xg for 20 min at room temperature to collect PRP and red blood cells separately. The PRP was centrifuged at 1,600xg for 15 min at room temperature to collect platelets. The red blood cells and platelets from healthy subjects were mixed with plasma from LVAD patients to reconstitute whole blood with a final platelet counts of 2x10^6^/µl and a hematocrit of 35%. The reconstituted blood was incubated with the fluorescent dye mepacrine (20µg/ml, Sigma Aldrich) for 5 min at room temperature to label platelets immediately before perfusion through the microfluidic chamber. This experiment allowed us to define the adhesive activity of VWF found in cryostored plasma samples from patients on LVAD supports without the confounding influence of erythrocytes and platelets from patients.

**Tail bleeding time**

We used tail bleeding time to determine the state of hemostasis in VWF deficient mice at baseline and after infusion with VWF before and after shear exposure (8,19). An anesthetized mouse was transected at 1/10 distance from the tail tip. The tail was immediately submerged in 50 ml of 37°C PBS. The PBS was collected for RBC counts using a whole blood analyzer and was also centrifuged at 4,000 rpm for 5 min at room temperature. The pellets were treated with 2 ml of the red blood cell lysis buffer (Sigma-Aldrich) for 10 min at room temperature. Following centrifugation at 10,000xg for 5 min, cell-free hemoglobin (Hb) in the supernatant was measured at OD 550 nm in a plate reader (Molecular Device).

**
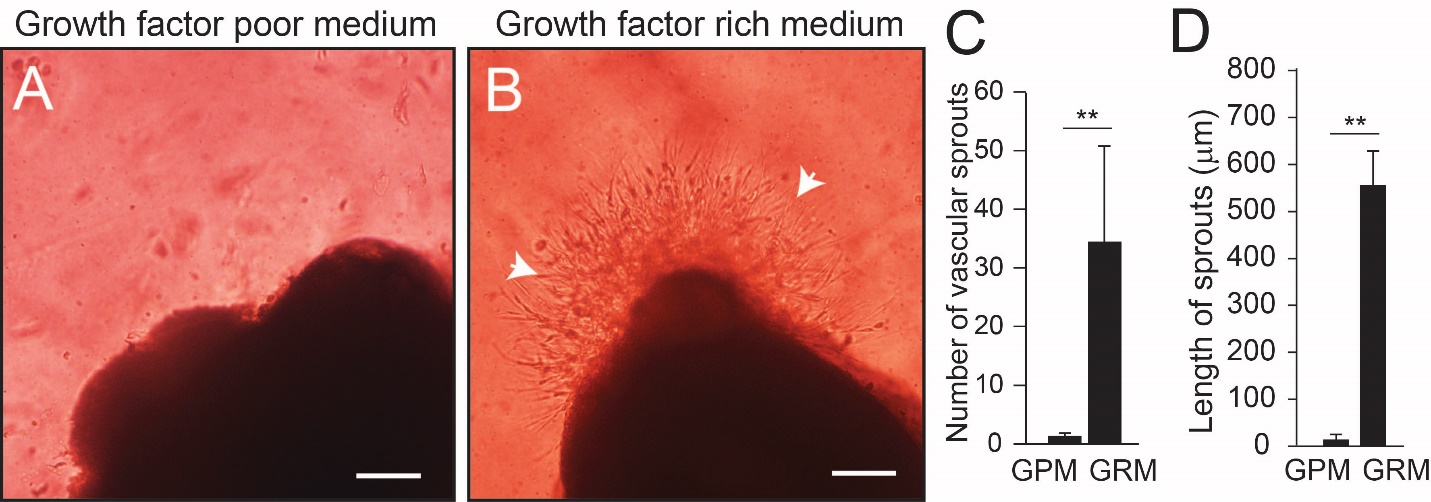
Supplemental Figure 1: In vitro angiogenesis assay:** Aortic arteries were dissected from C57BL/6J mice, cut into aortic vascular segments (AVSs) of 2 mm thick, and grown in Matrigels that contained either growth factor-poor medium (GPM, **A**) or growth factor-rich medium (GRM, **B**) for 14 days with daily medium changes (bar = 100 µm). On day 14^th^, the AVS grafts embedded in the Matrigel were observed under an invert-stage microscope. Numbers (**C**) and lengths (**D**) of vascular sprouts from AVSs were quantified from 6 independent experiments (paired t test, **p<0.01). This experiment set the baseline for studying EV-induced angiogenesis and a role of VWF in the process, as discussed in the main manuscript.


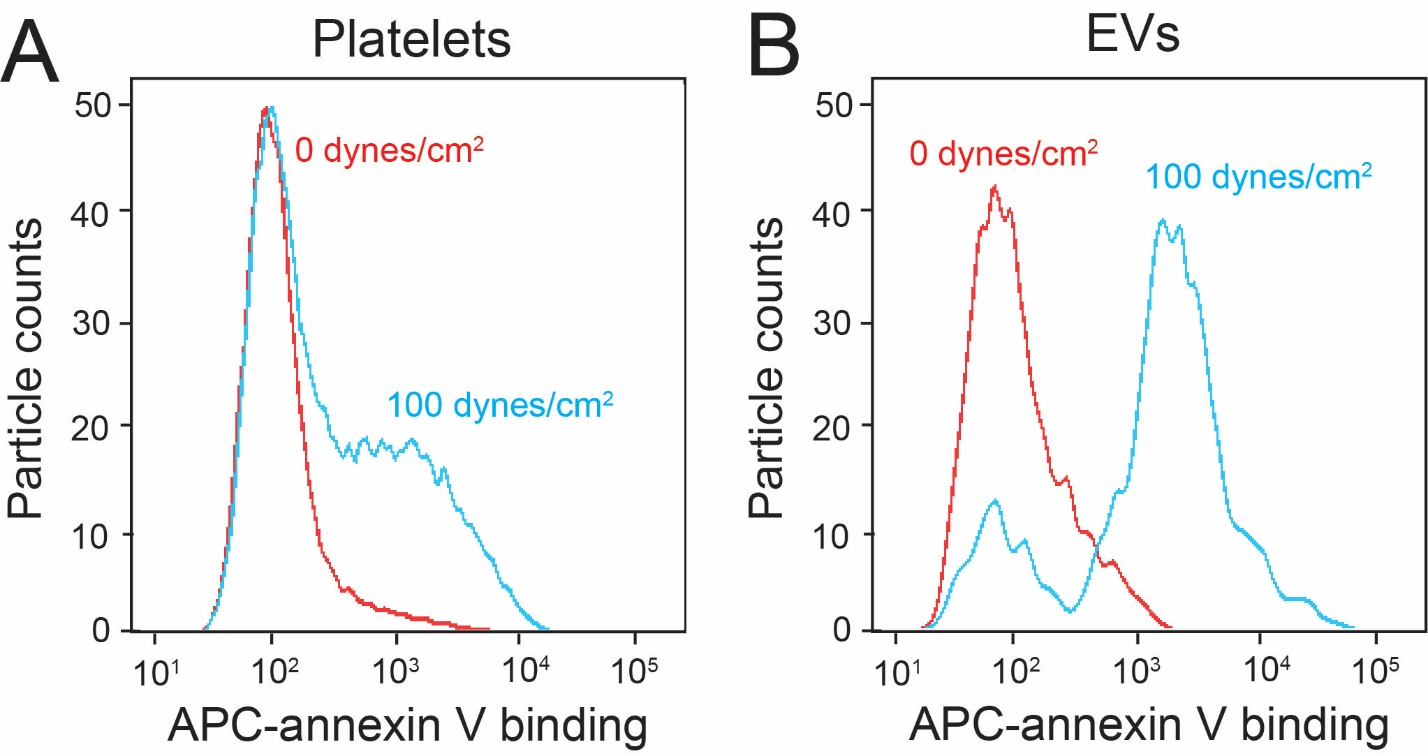


**Supplemental Figure 2:** Platelets from healthy subjects expressed anionic phospholipids (**A**) and released PS^+^ pEVs (**B**) after exposure to 110 dynes/cm^2^ of shear stress for 5 min at 37^o^C (representative plots from independent experiments of 10 subjects).


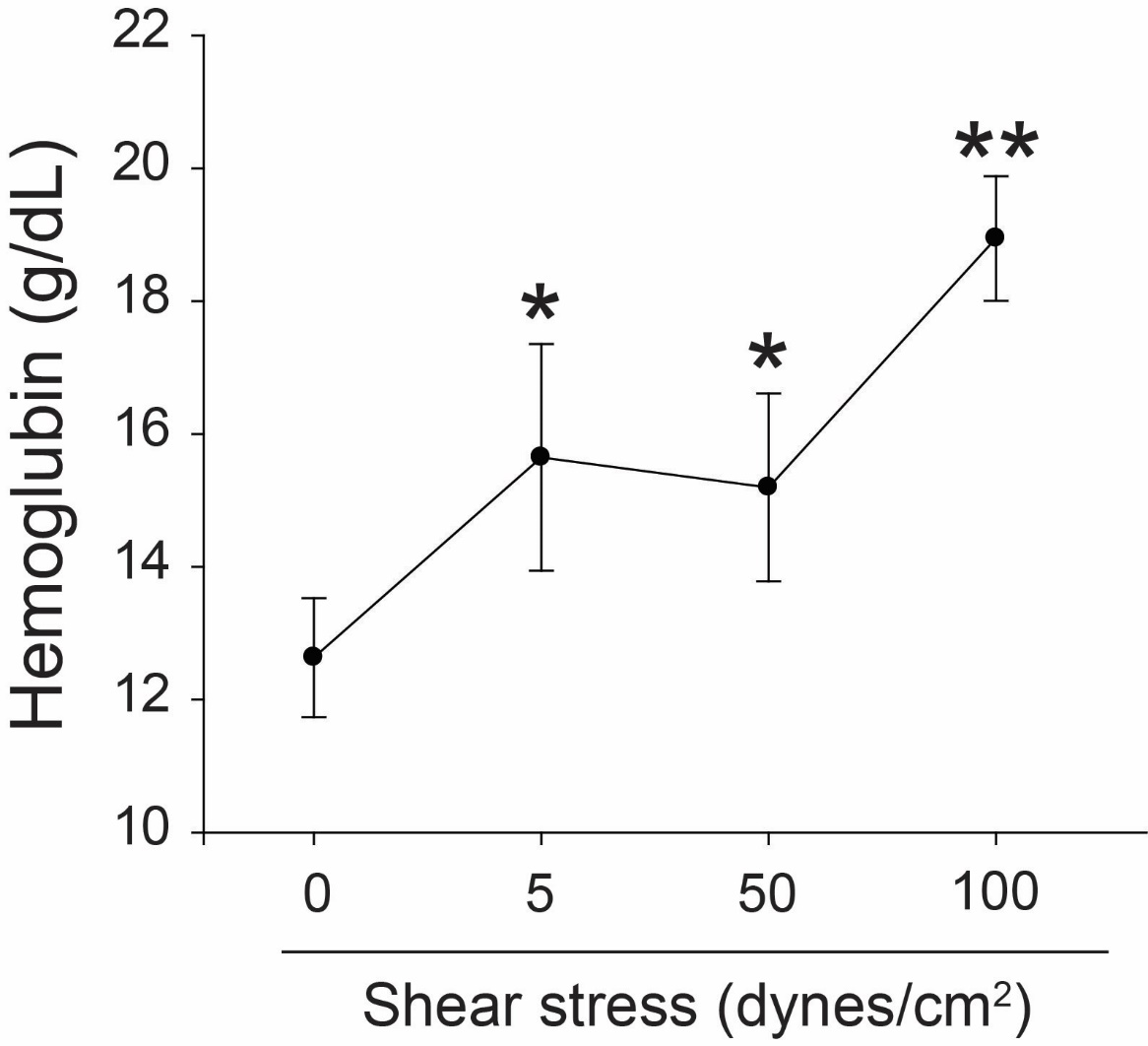


**Supplemental Figure 3:** Whole blood from healthy subjects underwent hemolysis detected by cell-free hemoglobin after exposure to shear stresses found in veins (5 dynes/cm^2^), small arteries (50 dynes/cm^2^), and LVAD-driven blood flow (110 dynes/cm^2^) on a cone-plate viscometer set at the control stress condition (n=9/stress level, one-way ANOVA, *p<0.05 vs. 0 shear, **p<0.01 vs. 50 and 110 dynes/cm^2^). The blood viscosities ranged from 5.71 to 6.92 cp among samples from the 9 subjects.


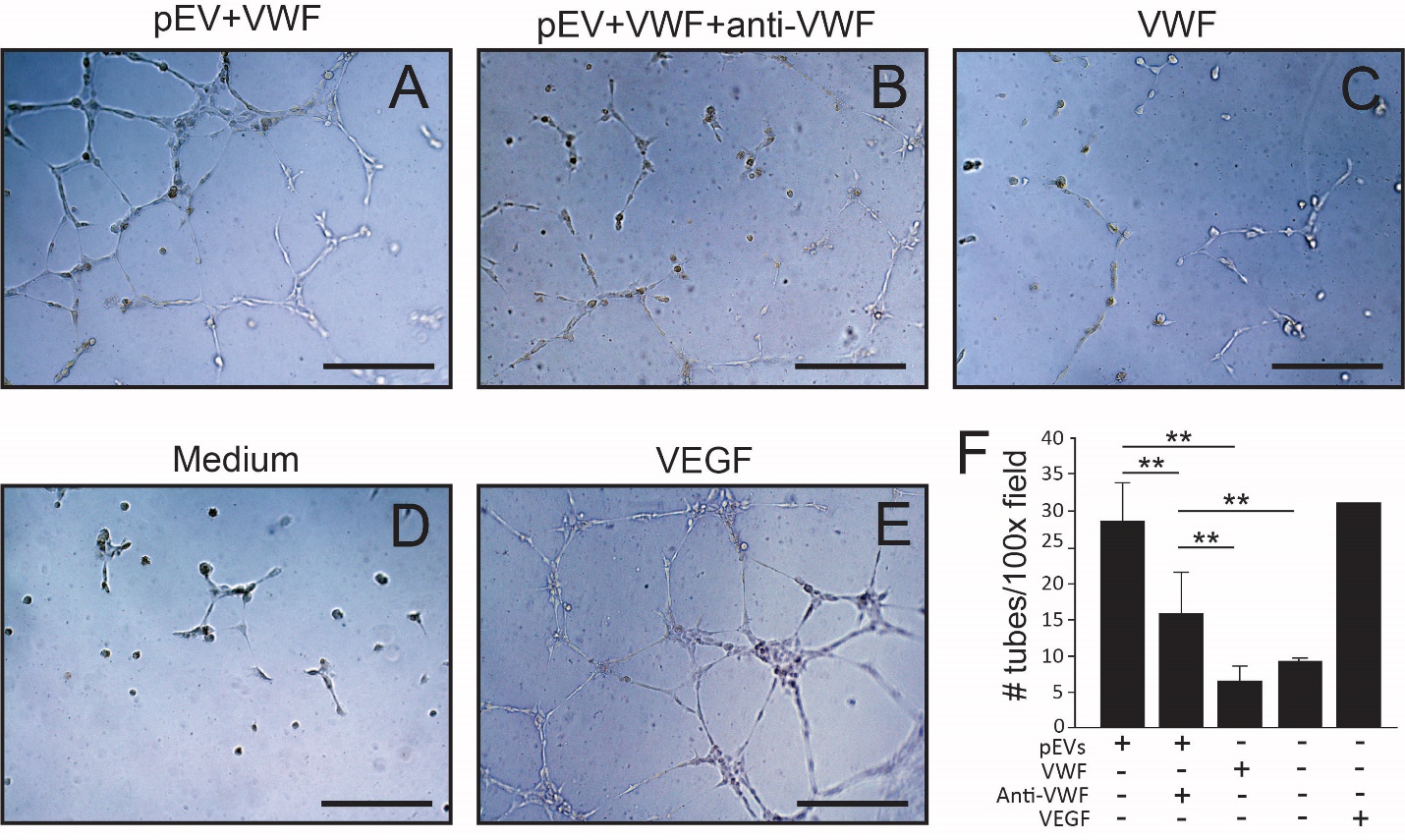


**Supplemental Figure 4:** The network formation assay detects the ability of endothelial cells to form vascular lumen on 2D-matrix gels. The panels A-C show representative images of endothelial vascular networks formed in EC cultures stimulated with (**A**) VWF^+^/CD41a^+^ pEVs, (**B**) pEVs with the VWF blocking antibody, (**C**) purified VWF (bar=200 um). Control cells were cultured in basic medium alone (**D**) and with VEGF (**E**). The panel **F** is the summary of multiple experiments (n=7/group, one-way ANOVA, **p<0.01)


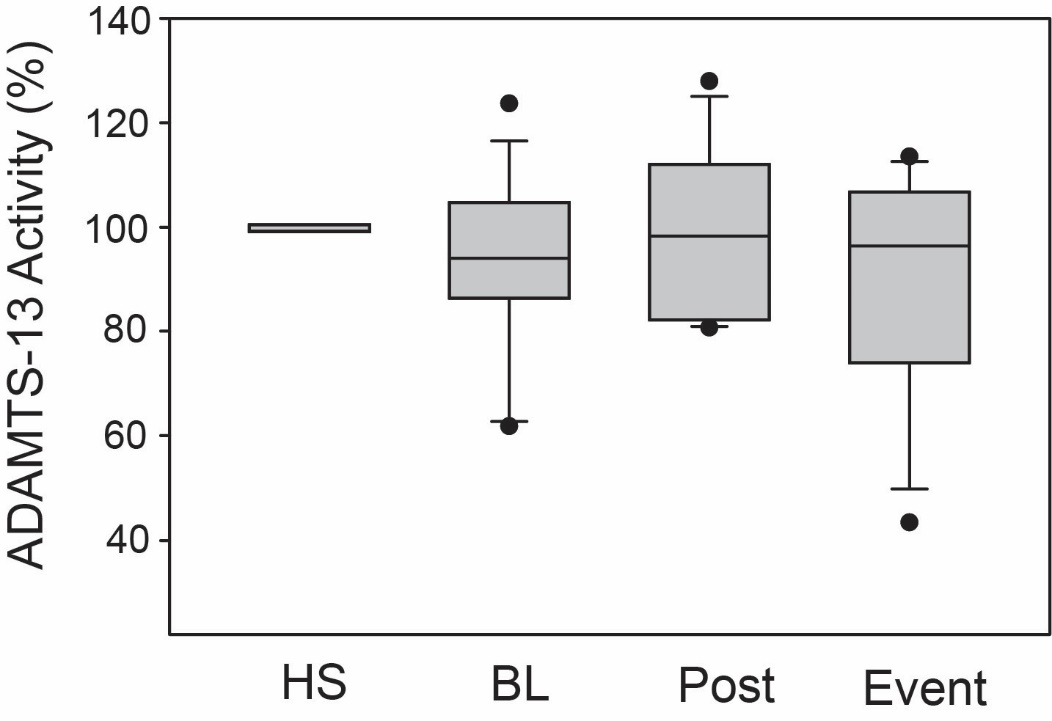


**Supplemental Figure 5:** ADAMTS-13 activity in LVAD patients and healthy subjects. It was measured by the ability of citrated plasma from patients and controls to cleave the short recombinant VWF peptide of 73 amino acids using a commercial FRET 73 assay, as previously reported (20).


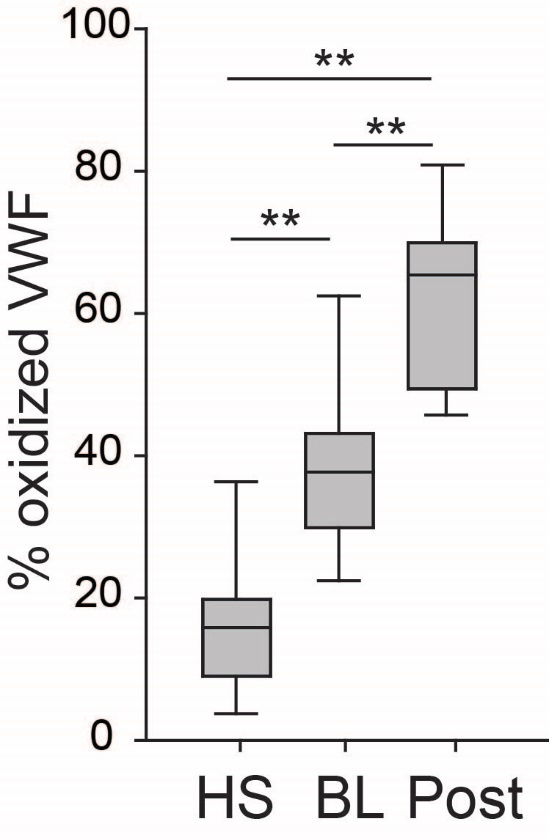


**Supplemental Figure 6.** VWF in plasma samples from healthy subjects (HS), patients at baseline (BL), and after LVAD implants (Post) were measured after thiol-containing VWF was depleted by the maleimide-PEO2-biotin coupled sepharose-streptavidin beads (n=26 patients and 13 healthy controls, one-way ANOVA, **p<0.01).

**
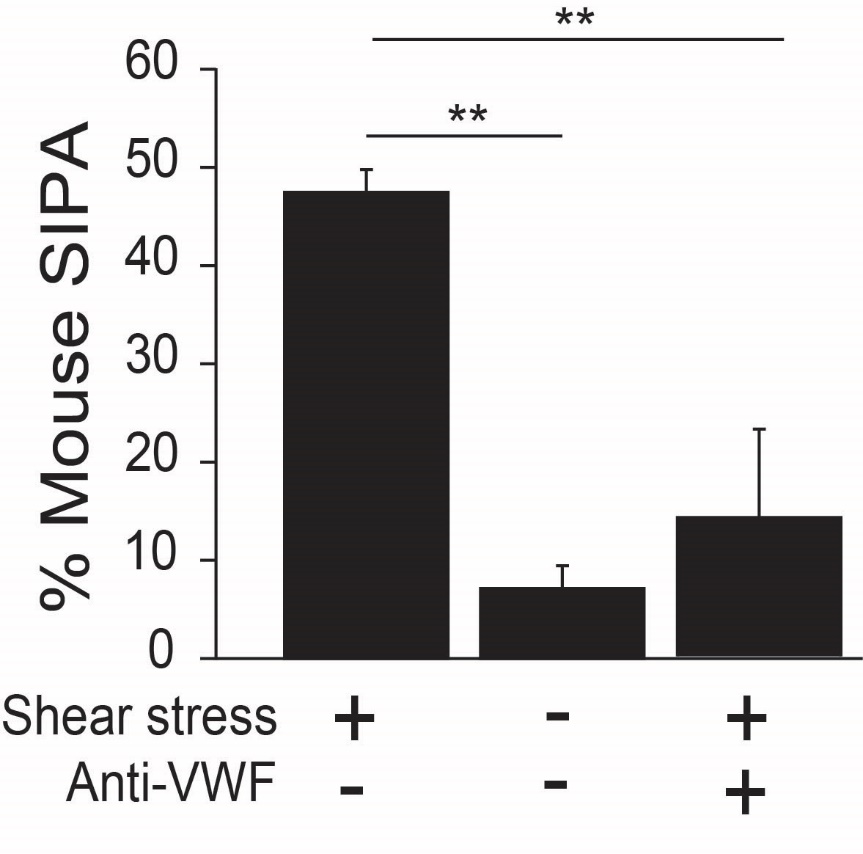
**

**Supplemental Figure 7.** PRP collected from C57BL/6J mice using 0.32% of sodium citrate as anticoagulant (final concentration) was exposed to 110 dynes/cm^2^ of shear stress for 5 min at 37^o^C. SIPA was measured using a particle counter (n=5/group, one-way ANOVA, **p<0.01).


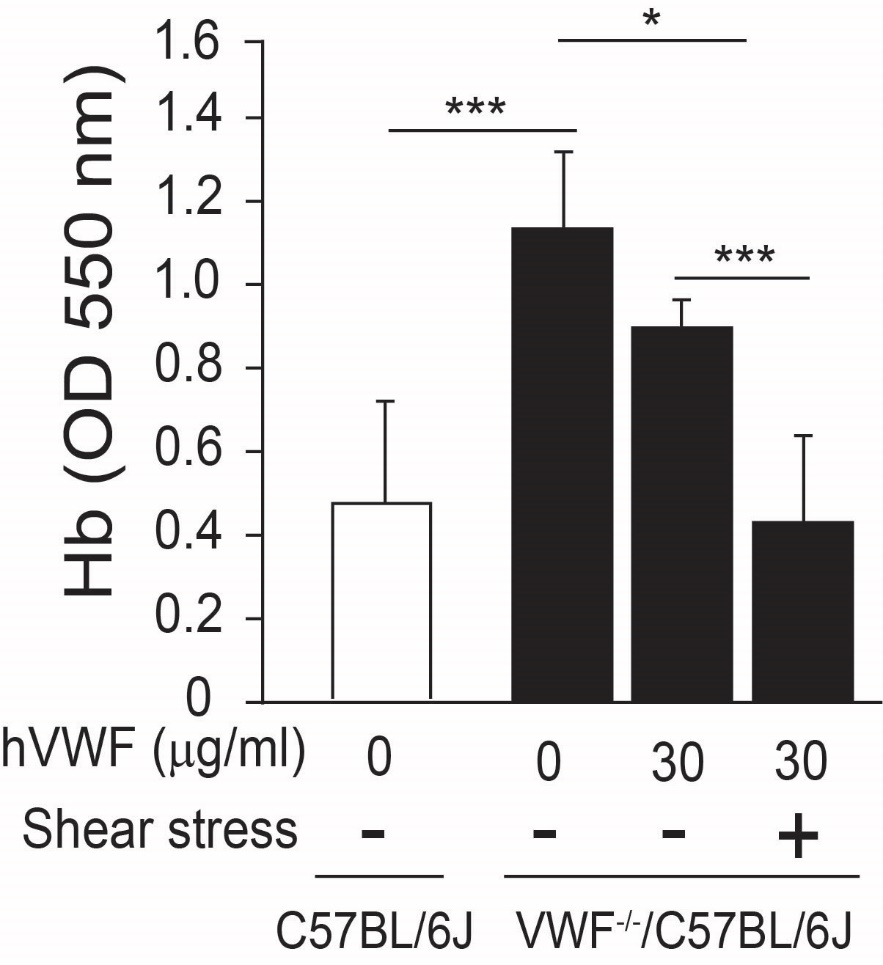


**Supplemental Figure 8:** Tail bleeding measured by levels of hemoglobin (Hb) in C57BL/6J mice and VWF deficient mice on C57BL/6J background, after infusion of purified human VWF before and after exposure to 110 dynes/cm^2^ of shear stress for 5 min at 37^o^C or an equal volume of saline (n=6/group, One-way ANOVA, *p<0.05, ***p<0.001).

**Supplemental References**

1. Campos M, Sun W, Yu F et al. Genetic determinants of plasma von Willebrand factor antigen levels: a target gene SNP and haplotype analysis of ARIC cohort. Blood 2011;117:5224-5230.

2. Campos M, Buchanan A, Yu F et al. Influence of single nucleotide polymorphisms in factor VIII and von Willebrand factor genes on plasma factor VIII activity: the ARIC Study. Blood 2012;119:1929-34.

3. Dong JF, Moake JL, Nolasco L et al. ADAMTS-13 rapidly cleaves newly secreted ultralarge von Willebrand factor multimers on the endothelial surface under flowing conditions. Blood 2002;100:4033-4039.

4. Arya M, Anvari B, Romo GM et al. Ultralarge multimers of von Willebrand factor form spontaneous high-strength bonds with the platelet glycoprotein Ib-IX complex: studies using optical tweezers. Blood 2002;99:3971-3977.

5. Xu X, Wang C, Wu Y et al. Conformation-dependent blockage of activated VWF improves outcomes of traumatic brain injury in mice. Blood 2021;137:544-555.

6. Gao C, Gong Z, Wang D et al. Hematoma-derived exosomes of chronic subdural hematoma promote abnormal angiogenesis and inhibit hematoma absorption through miR-144-5p. Aging 2019;11:12147-12164.

7. Nascimbene A, Hernandez R, George JK et al. Association between cell-derived microparticles and adverse events in patients with nonpulsatile left ventricular assist devices. The Journal of heart and lung transplantation : the official publication of the International Society for Heart Transplantation 2014;33:470-7.

8. Xu X, Wang C, Wu Y et al. Conformation-Dependent Blockage of Activated VWF Improved Outcomes of Traumatic Brain Injury in Mice. Blood 2020.

9. Wijeratne SS, Li J, Yeh H-C et al. Single-molecule force measurements of the polymerizing dimeric subunit of von Willebrand factor. Physical Review E 2016;93:012410.

10. Nascimbene A, Hilton T, Konkle BA, Moake JL, Frazier OH, Dong JF. von Willebrand factor proteolysis by ADAMTS-13 in patients on left ventricular assist device support. The Journal of heart and lung transplantation : the official publication of the International Society for Heart Transplantation 2017;36:477-479.

11. Choi H, Aboulfatova K, Pownall HJ, Cook R, Dong JF. Shear-induced disulfide bond formation regulates adhesion activity of von willebrand factor. JBiolChem 2007;282:35604-35611.

12. Dong JF, Berndt MC, Schade A, McIntire LV, Andrews RK, Lopez JA. Ristocetin-dependent, but not botrocetin-dependent, binding of von Willebrand factor to the platelet glycoprotein Ib-IX-V complex correlates with shear-dependent interactions. Blood 2001;97:162-168.

13. Tian Y, Salsbery B, Wang M et al. Brain-derived microparticles induce systemic coagulation in a murine model of traumatic brain injury. Blood 2015;125:2151-9.

14. Di Raimondo F, Azzaro MP, Palumbo GA et al. Elevated vascular endothelial growth factor (VEGF) serum levels in idiopathic myelofibrosis. Leukemia 2001;15:976-80.

15. McIlhenny C, George WD, Doughty JC. A comparison of serum and plasma levels of vascular endothelial growth factor during the menstrual cycle in healthy female volunteers. British journal of cancer 2002;86:1786-9.

16. Li Y, Choi H, Zhou Z et al. Covalent regulation of ULVWF string formation and elongation on endothelial cells under flow conditions. JThrombHaemost 2008;6:1135-1143.

17. Yuan H, Houck KL, Tian Y et al. Piperlongumine Blocks JAK2-STAT3 to Inhibit Collagen-Induced Platelet Reactivity Independent of Reactive Oxygen Species. PloS one 2015;10:e0143964.

18. Houck KL, Yuan H, Tian Y et al. Physical proximity and functional cooperation of glycoprotein 130 and glycoprotein VI in platelet membrane lipid rafts. Journal of thrombosis and haemostasis : JTH 2019;17:1500-1510.

19. Wu Y, Liu W, Zhou Y et al. von Willebrand factor enhances microvesicle-induced vascular leakage and coagulopathy in mice with traumatic brain injury. Blood 2018;132:1075-1084.

20. Kokame K, Nobe Y, Kokubo Y, Okayama A, Miyata T. FRETS-VWF73, a first fluorogenic substrate for ADAMTS13 assay. British journal of haematology 2005;129:93-100.
